# Oligonucleotide Sequence Motifs as Nucleosome Positioning Signals

**DOI:** 10.1371/journal.pone.0010933

**Published:** 2010-06-03

**Authors:** Clayton K. Collings, Alfonso G. Fernandez, Chad G. Pitschka, Troy B. Hawkins, John N. Anderson

**Affiliations:** 1 Department of Biological Sciences, Purdue University, West Lafayette, Indiana, United States of America; 2 National Cancer Institute, National Institutes of Health, Bethesda, Maryland, United States of America; 3 Department of Medical and Molecular Genetics, Indiana University-Purdue University Indianapolis, Indianapolis, Indiana, United States of America; National Institute on Aging (NIA), National Institutes of Health (NIH), United States of America

## Abstract

To gain a better understanding of the sequence patterns that characterize positioned nucleosomes, we first performed an analysis of the periodicities of the 256 tetranucleotides in a yeast genome-wide library of nucleosomal DNA sequences that was prepared by *in vitro* reconstitution. The approach entailed the identification and analysis of 24 unique tetranucleotides that were defined by 8 consensus sequences. These consensus sequences were shown to be responsible for most if not all of the tetranucleotide and dinucleotide periodicities displayed by the entire library, demonstrating that the periodicities of dinucleotides that characterize the yeast genome are, in actuality, due primarily to the 8 consensus sequences. A novel combination of experimental and bioinformatic approaches was then used to show that these tetranucleotides are important for preferred formation of nucleosomes at specific sites along DNA *in vitro*. These results were then compared to tetranucleotide patterns in genome-wide *in vivo* libraries from yeast and *C. elegans* in order to assess the contributions of DNA sequence in the control of nucleosome residency in the cell. These comparisons revealed striking similarities in the tetranucleotide occurrence profiles that are likely to be involved in nucleosome positioning in both *in vitro* and *in vivo* libraries, suggesting that DNA sequence is an important factor in the control of nucleosome placement *in vivo*. However, the strengths of the tetranucleotide periodicities were 3–4 fold higher in the *in vitro* as compared to the *in vivo* libraries, which implies that DNA sequence plays less of a role in dictating nucleosome positions *in vivo*. The results of this study have important implications for models of sequence-dependent positioning since they suggest that a defined subset of tetranucleotides is involved in preferred nucleosome occupancy and that these tetranucleotides are the major source of the dinucleotide periodicities that are characteristic of positioned nucleosomes.

## Introduction

The fundamental building block of the eukaryotic chromosome is the nucleosome, which consists of 147 bp of DNA, wrapped 1.65 times around an octamer of core histone proteins [reviewed in 1]–[3]. The histone octamer has been highly conserved throughout evolution and is composed of two copies of each histone (H2A, H2B, H3, and H4). Arginine and lysine residues on the surface of the octamer interact strongly with the negatively charged phosphate backbone of DNA ensuring that essentially any DNA sequence can be packaged into a nucleosome. However, during the past five years, large scale sequencing approaches and microarray hybridization technology have permitted the localization of the majority of nucleosomes in the genomes of yeast, worms, flies and humans, and these genome-wide studies have revealed that a surprisingly large fraction of nucleosomes are well-ordered with respect to their positions along the chromosomes [3]–[14]. These results are in agreement with a large body of earlier work, which has shown that nucleosomes are distributed in a nonrandom fashion along the eukaryotic chromosome [1]. The nonrandom positioning of nucleosomes along DNA in chromatin is thought to control access to regulatory proteins and is thus considered to be of fundamental importance in the regulation of the eukaryotic genome. [for reviews], [ see 1,15]. Consequently, elucidation of the factors that govern nucleosome positioning is required for a better understanding of genome regulation.

The positioning of nucleosomes depends on two fundamental factors. First is the DNA sequence preference, but it is uncertain as to what fraction of nucleosomes is positioned by sequence alone *in vivo* [12], [13], [16], [17]. Second are epigenetic factors including ATP-dependent chromatin remodeling factors, DNA methylation, posttranslational modification of histones and DNA bound regulatory proteins [18]–[21]. It is likely that DNA sequence dictates the ground state for the ordering of nucleosome positioning, and that epigenetic factors are superimposed over this state for determination of the final architecture and function of chromatin in the cell [14], [22]. The observation that the chromatin structures of most, but not all, promoters in yeast are maintained throughout the cell cycle seems to point to the importance of the both the primary DNA sequence and epigenetic factors in the control of gene regulation [23].

DNA sequence is thought to direct the positioning of nucleosomes by two distinct mechanisms: the inhibition of nucleosome formation and the preferential assembly of the core particle. Homopolymeric stretches of dA: dT that are >10–20 bp in length represent an important genomic feature that serves to inhibit nucleosome formation. Early studies demonstrated that these tracts are stiff and resistant to bending forces, and this property has long been associated with their ability to inhibit nucleosome formation *in vitro* and *in vivo* [24], [25]. These sequences produce gaps between nucleosomes, and the nucleosome-free regions (NFRs) are hypersensitive to nuclease probes such as DNase 1. These gaps are frequently found in constitutively active promoters where they have been viewed as entry sites for the transcriptional machinery. Chromatin gaps that arise from these sequence elements have also been implicated in the control of replication, initiation, and transcription termination. In fact, the long homopolymeric dA∶dT tracts have been considered to represent major determinants of nucleosome organization in all eukaryotes [reviewed in 26].

The second mechanism by which DNA directs the organization of nucleosomes involves sequences that facilitate nucleosome formation and stability and promote positioning of the histone octamer at single genomic sites. These positioning sequences were originally obtained from a wide range of eukaryotes and their viruses, and it is now suspected that they are more widespread in the eukaryotic genome than was originally anticipated [1], [3]. These positioning elements frequently occur in the vicinity of promoters and enhancers, and a variety of direct functional studies provide strong emerging evidence that they directly regulate transcriptional initiation and other genomic functions as well. Recent studies have suggested that these sequences are preferentially associated with variable promoters rather than constitutively active ones, and it has been suggested that they render these promoters susceptible to epigenetic regulation [27]. However, the precise mechanism by which these elements facilitate nucleosome positioning and stability is not known.

During nucleosome formation, a relatively stiff DNA molecule is tightly wound around the histone octamer resulting in a DNA conformation that is highly strained. According to one widely accepted view, certain dinucleotide sequence patterns along the length of nucleosomal DNA can best relieve this strain by enhancing anisotropic flexibility, and these sequences should therefore be preferentially packaged into nucleosomes. This concept was originally advanced by Trifonov and Sussman [28] and has been incorporated into a large number of models for predicting nucleosome positioning from AA/TT/TA sequence periodicities [7], [16], [29], [30]. However, to our knowledge, there is no direct experimental evidence that dinucleotide periodicities per se are involved in dictating preferred nucleosome occupancy, and it is becoming increasingly apparent that sequence-dependent structures of DNA in solution and in the nucleosome cannot be adequately described at the level of the dinucleotide. For example, curved DNA that arises from oligonucleotide length A-tracts arranged in a ∼10 bp periodicity is preferentially packaged into nucleosomes [31]–[34]. It was also pointed out long ago that dinucleotide analysis represents an oversimplification of the problem since the AA/TT repeat pattern in 177 chicken erythrocyte nucleosome fragments is largely due to AAA/TTT [35]. Recent studies with synthetic DNA fragments have also shown that certain oligonucleotide sequences containing TA steps function in conferring high nucleosome affinity and positioning activity *in vitro* and the two major experimentally identified core elements in this group are the tetranucleotides CTAG and the related sequence TTAA [36], [37]. These sequences have been identified at the same locations in a few natural nucleosome-positioning sequences [36]–[38], but there have been no systematic computational studies aimed at describing these sequences in genomic nucleosomal DNA. Taken together, these results seem most consistent with an oligonucleotide model for nucleosome positioning.

Proteins that bind in the DNA major groove most often utilize a direct readout strategy for the recognition of nucleotide sequences that involves hydrogen bonding between DNA bases and amino acid residues. In contrast, proteins like histones that interact in the minor groove often utilize indirect modes of recognition, which are dependent on intrinsic shapes, and mechanical properties of the DNA [39], [40]. The informational content of DNA that is used for assessment of indirect readout mechanisms depends on the sequence length. The relative frequencies of A-T vs. G-C bp provides information on DNA stability which has been used for characterization of DNA in solution and in the nucleosome [41]. However, dinucleotides of the same composition can display markedly different characteristics as exemplified by the divergent properties of AA, AT and TA [42]–[44]. The smallest units of DNA that contain sequence information are the 16 dinucleotides and characterizations of DNA structure at this level have yielded important insights into the factors which affect the conformational properties of DNA and its packaging into nucleosomes. However, dinucleotide steps are sensitive to their immediate sequence context as suggested by the observations that the conformational properties of the dinucleotide YR in the tetranucleotide XYRZ is dependent on the identity of X and Z [45], [46]. Several other sequences that have well-defined conformational properties at the dinucleotide level are so strongly affected by their neighbors that they behave completely different at the tetranucleotide level [47]. For this reason, the 256 tetranucleotides have most recently been the subject of investigation, and the characterization of these units in terms of flexibility, stability and minor groove widths have now been reported [47], [48].

In this study, we characterized tetranucleotides in positioned nucleosomes in genome wide libraries from yeast and *C. elegans*. The overall aims were to identify tetranucleotide periodicities that are likely to be associated with the sequence-dependent positioning of nucleosomes and to compare the importance of these tetranucleotides to dinucleotides periodicities in the control of nucleosome placement. The results suggested that a defined subset of tetranucleotides is involved in preferred nucleosome occupancy and that these tetranucleotides are the major source of the dinucleotide periodicities that are characteristic of positioned nucleosomes.

## Results

### Analysis of Tetranucleotides in the Yeast *In Vitro* Nucleosome Library

The yeast genome-wide library of nucleosome sequences described by Kaplan et al. [12] was used to provide a description of the tetranucleotide sequence patterns in *in vitro* positioned nucleosomes. The library was prepared by high-salt reconstitution methods using purified histones from chicken erythrocytes and naked high molecular weight yeast DNA using a DNA: histone mass ratio of 2.5∶1. Reconstituted chromatin was then digested with micrococcal nuclease (MNase) and the nucleosome core particle DNAs were sequenced by utilization of the Illumina Solexa technology. The DNA excess should select for preferred histone octamer binding sequences in the absence of epigenetic factors and minimize the possibility that a nucleosome positioned by sequence does not serve to position adjacent nucleosomes by a sequence-independent boundary effect.

To our knowledge, there have been no systematic characterizations of the DNA sequence periodicities in positioned nucleosomes at the tetranucleotide level. As a first step aimed to address this problem, the frequency profiles of the 256 tetranucleotides were examined in the yeast library. Fourier-transform analysis of this frequency data was used to construct Table S1, which gives the periodicities, fractional variations of occurrence (FVOs), and phase angles for each tetranucleotide. The FVO represents the strength of the periodic oscillations in a frequency profile relative to the frequency average. The phase angle is used to indicate whether the minor groove of a tetranucleotide faces inward or away from the histone surface. In order to compare the characteristics of tetranucleotides with dinucleotides, Table S2 was constructed which gives the corresponding dinucleotide parameters.

**Figure 1:**
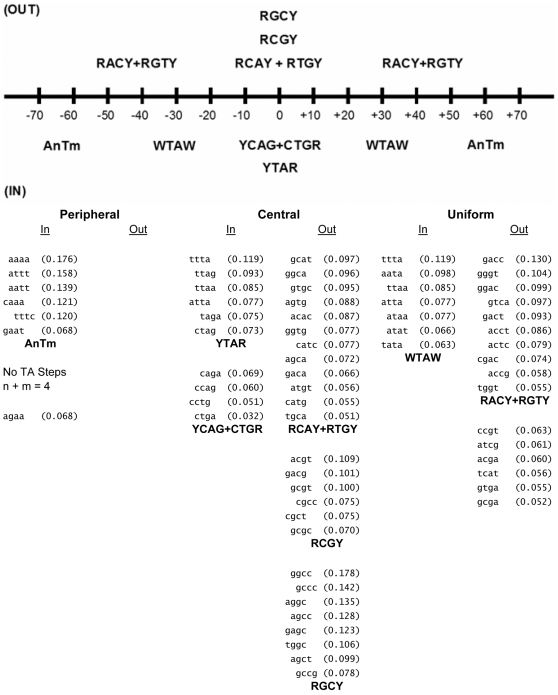
Analysis of tetranucleotides in the *in vitro* library. The 63 tetranucleotides from the Kaplan et al. 2009 *in vitro* replicate 1 library [12] that satisfied the criteria described in the text are grouped according to preferential distribution along nucleosome DNA (peripheral, central, or uniform) and rotational orientation of the DNA minor groove (In vs. Out). The numbers beside the tetranucleotides are FVO_10.2_ values. In each column, the tetranucleotides are grouped according to similarities in sequence. Consensus sequences derived from the 8 groups are presented in bold face type below the tetranucleotides. The map at the top depicts the general positions of the consensus sequences and their rotational orientation. The sequence AnTm represents tetranucleotides without TA steps where n+m = 3 or 4. Y = Pyrimidine, R = Purine.

A four-step procedure was performed in order to simplify the presentation of the tetranucleotide data in Table S1. First, approximately 30% of the tetranucleotides were omitted because they displayed weak ∼10 bp periodicities and consequently low FVOs (Figure S1). Second, only those tetranucleotides that had minor grooves facing the histone octamer (phase angles <−135° or >+135°) or away from the octamer (phase angles >−45° or <+45°) were considered for further study. These angles were chosen because we assume that sequence-dependent bending, bendability, and kinking would most likely depend on sequences with these rotational orientations. Third, only unique tetranucleotides were considered for the analysis. This simplification was justified because unique tetranucleotides shared identical FVOs and opposite phase angles with their reverse complements (Table S1). A total of 63 tetranucleotides satisfied these three criteria and are displayed in Figure 1. A final distinction was made according to the relative frequency along the nucleosomal DNA. The tetranucleotides were classified as peripherally located, centrally located or uniformly distributed along the sequence. File S1 shows the frequency profiles of the tetranucleotides, providing examples of these distributions.

Characteristics of the 63 tetranucleotides that satisfy the above criteria are presented in Figure 1. Tetranucleotides are grouped according to preferential distribution along nucleosome DNA (peripheral, central, or uniform) and rotational orientation of the DNA minor groove (In vs. Out). The numbers beside the tetranucleotides are FVOs. In each column, the tetranucleotides are grouped according to similarities in sequence. Consensus sequences derived from the 8 groups are presented in bold face type below the tetranucleotides. Each tetranucleotide in a group shared a common dinucleotide and at least one common 5′ or 3′ flanking base. If the common dinucleotide was in the center of the tetranucleotide, both flanking bases were required to match the consensus sequence. The permissible 1bp staggers in the alignment procedure roughly correspond to the assigned phase angle ranges of +/−45 degrees. The map at the top of the table depicts the general positions of the consensus sequences and their rotational orientation. Seven of the 63 tetranucleotides could not be described by a consensus sequence.

**Figure 2:**
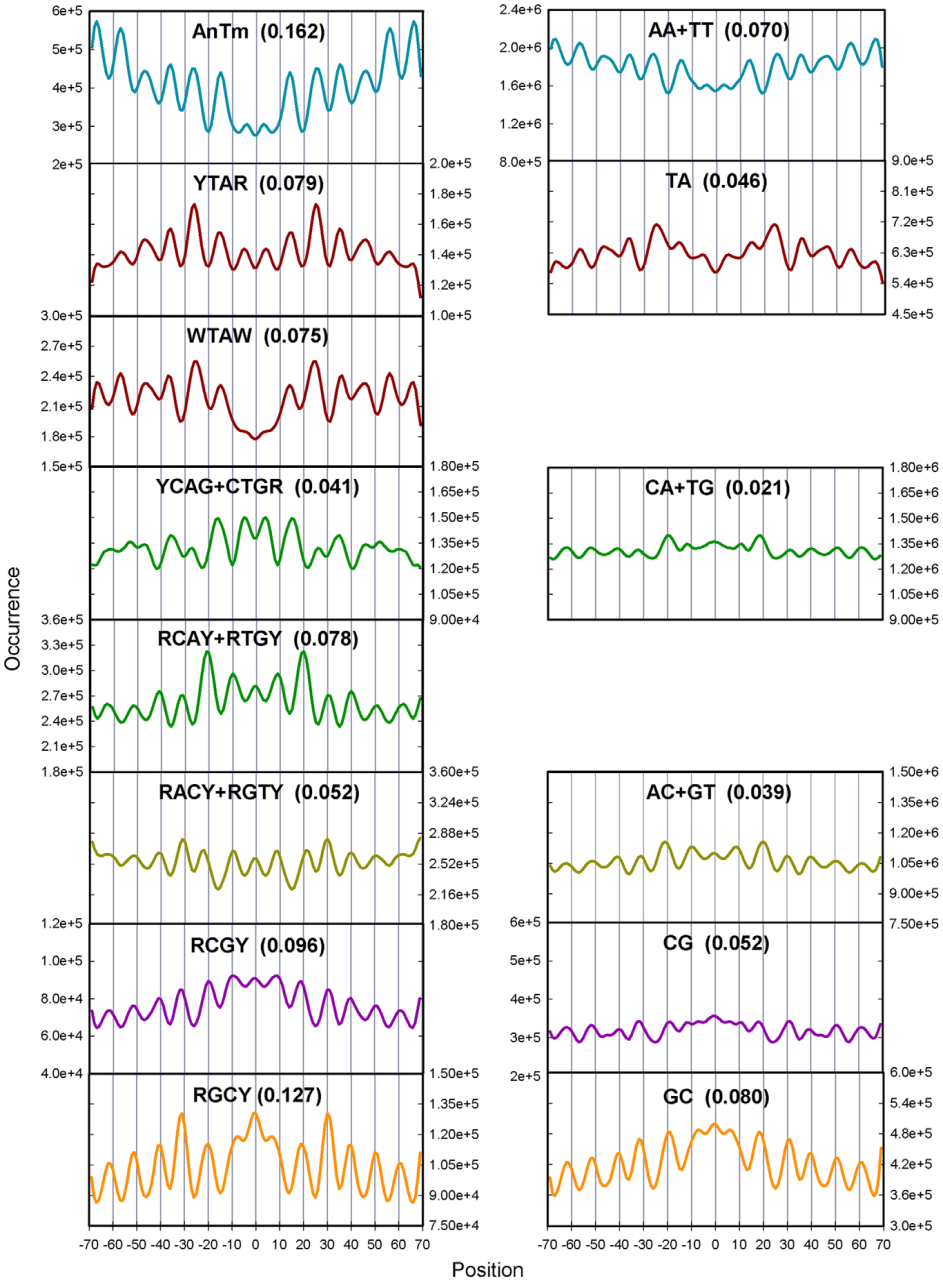
Frequency profiles generated by the tetranucleotide consensus sequences from **Figure 1**. The sums of the occurrences of all tetranucleotides in the Kaplan et al. 2009 *in vitro* replicate 1 library [12] that make up the consensus sequences from Figure 1 are given in the left panels of the figure. The profiles of the corresponding central dinucleotide sequences of the tetranucleotides are shown in the right panels. The numbers adjacent to the sequence designation in parentheses are the FVO_10.2_ values.

Figure 2 (Left panels) shows the occurrences of the consensus tetranucleotide sequences derived from the studies in Figure 1. For comparison, the profiles of the corresponding central dinucleotide sequences of the tetranucleotides are shown in the right panels to illustrate the importance of the central flanking bases. The numbers adjacent to the sequence designation in parentheses are the FVOs. The results with the tetranucleotides are in complete agreement with those in Figure 1 in terms of peripheral inward localization of AnTm, the central inward localization of YTAR and YCAG/CTGR, the uniform inward localization of WTAW, the uniform outward localization of RACY/RGTY, and the central outward positioning of RCAY/RTGY, RCGY and RGCY. The strengths of 10.2 bp periodicities, as measured by the FVO values, are, on average, 1.8-fold higher for the tetranucleotide consensus sequences than the corresponding dinucleotide sequences.

**Figure 3:**
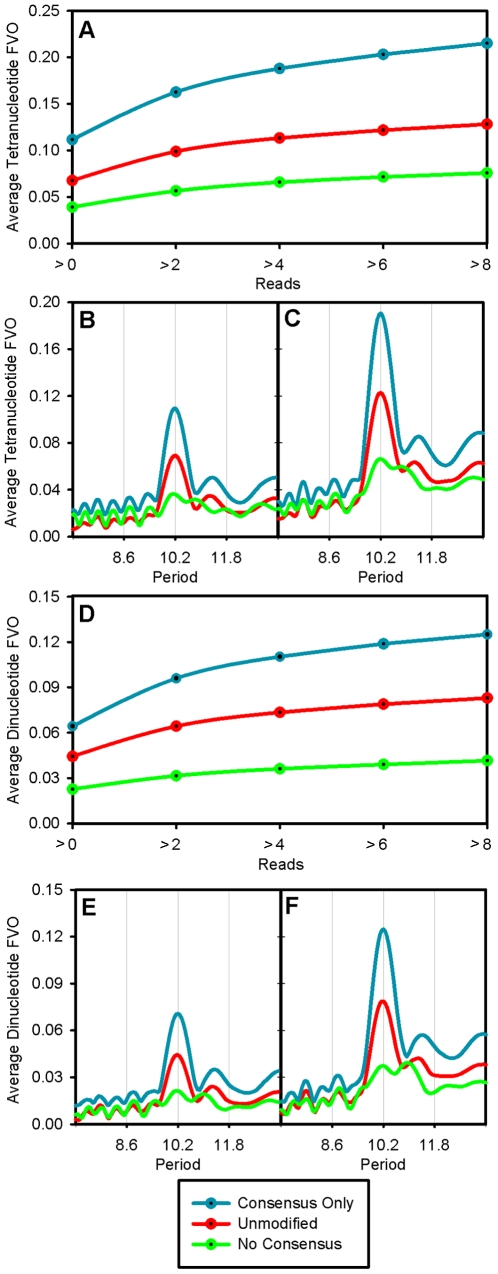
Contribution of the consensus tetranucleotides to the average tetranucleotide and dinucleotide periodicities. In order to evaluate the significance of the consensus tetranucleotides, the Kaplan et al. 2009 *in vitro* replicate 1 library [12] (Unmodified Library) was modified as described in the Methods Section to yield the Consensus Only and No Consensus libraries. The average FVO of the tetranucleotides for a 10.2 bp periodicity in the three libraries as a function of reads is given in Panel A. Panels B and C present graphs of the average tetranucleotide FVO versus period for all reads and for sequences with greater than 6 reads, respectively, for each of the three libraries. An analogous study was performed on the dinucleotides, which is represented by Panels D–F.

There are a total of 136 unique tetranucleotides, which include the 24 tetranucleotides that make up the 8 consensus sequences. The remaining 112 unique tetranucleotides were not analyzed in Figure 2 because they had low FVOs, intermediate phase angles and/or because they did not align to the consensus sequences. The analysis in Figure 3A–C was carried out to ascertain the relative contribution of the 24 consensus tetranucleotides to the strength of the ∼10 bp tetranucleotide periodicities in the entire library. In this analysis, we compared the average strength of the periodicities of all tetranucleotides in the *in vitro* library to the strength of the periodicities of tetranucleotides in a modified library that lacked the tetranucleotide consensus sequences and to another library that contained only isolated and overlapping consensus tetranucleotides. The original spacing of the tetranucleotides in the two modified libraries was retained by using the procedure described in the Materials and Methods Section. In panel A, the strengths of the periodicities in the three sequence sets were examined as a function of increasing sequence reads since nucleosome sequences that are characterized by a higher number of reads presumably correspond to DNA sequences with higher affinity for the histone octamer and/or higher positioning activity. Panels B and C illustrate the nature of the periodicities in the three libraries for all reads and for sequences with greater than 6 reads, respectively. The strength of the 10.2 bp periodic signal increased with increasing numbers of reads with the unmodified library. This increase was substantially greater with the library consisting of only consensus sequence tetranucleotides, which is expected of sequence elements that are responsible for nucleosome positioning. In contrast, the periodic signal was greatly reduced (>80%) with the library containing only the non-consensus sequence tetranucleotides, which shows that the consensus sequence tetranucleotides are the major contributors to the periodic pattern of the entire library.

A similar approach was used to assess the contribution of the 24 consensus tetranucleotides to the average of the dinucleotide periodicities in the *in vitro* library (Figure 3 D–F). The results show that there is a near complete loss of dinucleotide periodicity in the absence of the tetranucleotides that comprise the consensus sequences. In addition, there was essentially a complete loss of the periodic patterns displayed by each of the ten unique dinucleotides in the library that lacked the 24 consensus sequence tetranucleotides as shown in Figure S2. These observations demonstrate that the periodicity of dinucleotides in the *in vitro* library are, in actuality, due primarily or exclusively to the tetranucleotides (or longer oligonucleotides) that comprise the 8 consensus sequences.

### Relationships between Tetranucleotides and Nucleosome Stability and Positioning

**Figure 4:**
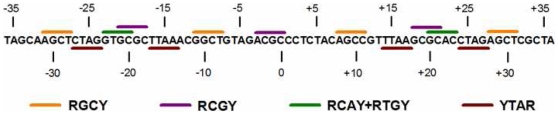
Tetranucleotide consensus sequences in the PCR SELEX conserved central region. The sequence shown in the figure is the conserved central region obtained by the SELEX approach for high affinity nucleosome binding sequences and is from reference 50. Indicated in the figure are four of the tetranucleotide consensus sequences from Figure 2 and the corresponding tetranucleotides that were identified in multiple locations along the 73 bp sequence.

Synthetic DNA fragments which display high affinity for the histone octamer *in vitro* were used to study the relationships between the consensus tetranucleotide sequences in Figures 1 and 2 and nucleosome stability and positioning activity. The first sequence set was prepared by a SELEX approach starting with a large pool of chemically synthetic random DNA molecules [49], [50]. These fragments display the highest reported affinities for the histone octamer, and this characteristic likely arises from multiple sequence determinants. The 73 bp central regions of these sequences, which contain all information needed for high nucleosome affinity, were used to derive the conserved sequence that is shown in Figure 4 [50]. Indicated along this sequence are tetranucleotide sequences that are represented by four of the tetranucleotide consensus sequences. These 13 tetranucleotides occupy over 60% of the length of the sequence and display the same rotational orientation as the corresponding tetranucleotide consensus sequences in the yeast genome-wide library. The positions of the 13 tetranucleotide sequences along this sequence also closely coincide with regions of high frequencies in the occurrence profiles shown in Figure 2. For example, the high occurrence peaks of YTAR at positions at +/−15 and +/−25 in the database sequences correspond to CTAG and TTAA at +/−15 and +/−25 in the synthetic sequence. Similar positional correspondence is seen with GTGC and GCAC (RCAY+RTGY), AGCT (RGCY), and GCGC (RCGY). The high frequency of these tetranucleotides in the synthetic fragment, as well as the similarities in rotational and translational positions within the nucleosome, argue for a functional role of these sequences in the control of nucleosome occupancy *in vitro*.

**Figure 5:**
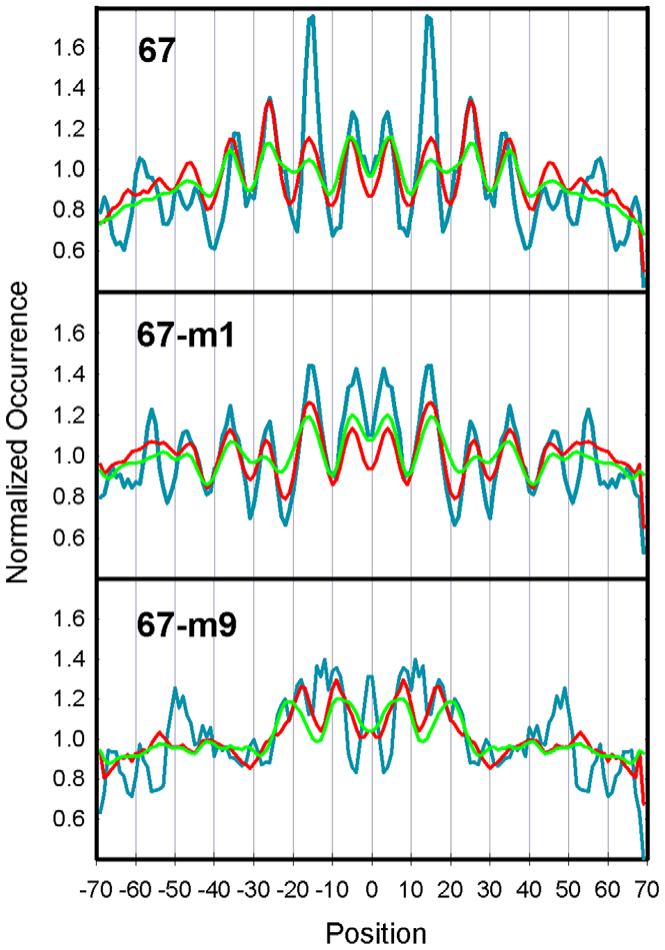
Analysis of Fragment 67 mutations in the *in vitro* library. The top panel shows an analysis of the occurrences of the nucleosome sequences in the yeast database that contains the 8 bp sequence CTCTAGAG that surrounds the KMnO4 hyperreactive T residue in Fragment 67 (blue lines). Also shown in this panel are occurrence profiles when the central 6 bp (red lines) and 4 bp (green) of this sequence were used in the analysis. All of the profiles were normalized by their average frequencies. The occurrences of the reverse complements were added to the frequency profiles for the 67-m1 sequences as well as all sequences in Table 1 that are non-palindromic. The average FVO_10.2_ value for all 65,536 octamer sequences was 0.112 with a SD of 0.059; therefore, the FVO of CTCTAGAG is more than 2 SD above the mean (Table 1). The same analysis was carried out for 67-m1 and 67-m9 where the central TA step was changed to TG/CA (middle panel) and flanking bases were exchanged from C and G to G and C (bottom panel).

A more detailed functional analysis of the single consensus sequence YTAR is given in Figure 5. A synthetic 223 bp DNA fragment known as 67 displays a high affinity for the histone octamer and positions a nucleosome at a single translational frame [36], [37]. The nucleosome that assembles onto Fragment 67 also contains a single site that is hypersensitive to KMnO_4_. The hyperreactive T residue is contained within a TA step, which is located 15 bp upstream from the dyad at a site that is highly distorted in the nucleosome. Mutational analysis revealed that both the TA step and its flanking bases are required for high affinity octamer binding and translational positioning. Figure 5 (top panel) shows an analysis of the occurrences of the nucleosome sequences in the yeast database that contains the 8 bp sequence CTCTAGAG that surrounds the hyperreactive T residue in Fragment 67. Also shown in this panel are occurrence profiles when the central 6 bp and 4 bp of this sequence were used in the analysis. The results revealed characteristic patterns of occurrences that are consistent with experimental data in that the frequencies of each sequence are highest within the central turns of the nucleosome, with the most prominent peaks at positions +/−15 and +/−25 from the dyad. The FVO value of CTCTAGAG was also >2 SD above the mean of all octamers in the yeast database (see legend) and its enrichment at the +/−15 bp region relative to the frequency average was >4 SD above the mean (data not shown). There was a reduction in the strength of this pattern when the central TA step was changed to TG/CA, and a near loss of the periodicity when the TA flanking bases were exchanged from C and G to G and C (middle and bottom panels). Corresponding reductions in nucleosome stability and positioning activity were seen when these mutations were made in Fragment 67 [37].

The analysis in Figure 5 was carried out for 16 mutations that were made in Fragment 67. These fragments were tested previously for stabilities (ΔG) and positioning activities relative to wild-type 67 (% 67) in *in vitro* nucleosome reconstitution assays [37]. The results of these published experiments are given in Table 1. The FVOs of the 67 derivative sequences were determined from the frequency profiles like those in Figure 5 (File S3). Only the octamer FVOs are shown in Table 1 while the FVOs of the hexamers and tetramers are provided in File S3. Changes in the TA step in fragment 67 resulted in reductions in nucleosome stability and positioning activity and these changes were accompanied by corresponding reductions in FVOs (37, Table 1A). The order for obtaining stable nucleosomes, positioning activity and FVO values was TA>TG>TT≥TC≈GG≈GA≈AT. Likewise, there were reductions in nucleosome stabilities and positioning activities with corresponding decreases in FVOs when TA flaking bases were altered (Table 1B).

**Table 1:** Correlations between nucleosome stability, positioning activity, and strength of sequence periodicities in the yeast *in vitro* nucleosomal DNA sequence Library.

	Construct	Sequence	ΔG	%67	FVO
**A**	67	CTCTAGAG	0	100	0.256
	67-m1	CTCCAGAG	586	85	0.233
	67-m2	CTCAAGAG	678	77	0.143
	67-m3	CTCCCGAG	956	69	0.083
	67-m4	CTCATGAG	1195	61	0.079
	67-m5	CTCGAGAG	974	69	0.018
	67-m6	CTCTCGAG	1129	61	0.018
**B**	67-m7	CTGTAGAG	556	84	0.139
	67-m8	CTCTACAG	556	82	0.139
	67-m9	CTGTACAG	761	68	0.031
	67-m10	CAGTACTG	1130	58	0.100
**C**	601+25	TGCTAGAG	31	96	0.143
	601−39	GACTAGGG	92	83	0.130
	5S-16	CTTTAAAT	−140	93	0.156
	601−16	GGTTAAAA	107	80	0.146
	5S-7	GCTTAACT	171	83	0.066
	601+16	GTTTAAGC	−247	88	0.183
**D**		**Length:**	**4**	**6**	**8**
	**r**	**ΔG vs FVO:**	−0.619	−0.818	−0.783
	**r**	**%67 vs FVO:**	0.755	0.827	0.616

FVO_10.2_ values for octamer sequences were determined from occurrence profiles like those in Figure 4. The stabilities (**Δ**G) and positioning activities relative to wildtype 67 (%67) are from reference 37. Correlation coefficients (r) are for tetramers (4), hexamers (6), and octamers (8).

KMnO_4_ hypersensitive TAs were also observed in other sequences that position nucleosomes at single translational sites including the synthetic fragment 601 and the 5S rDNA sequence from sea urchin [36], [37]. The 10 bp sequences surrounding these TA step conferred high stability and positioning activity when they replaced the −10 to −20 bp region in Fragment 67 [36], [37]. The major core sequences in this set were TTAA and CTAG with the consensus YTAR. Most of these sequences were located at approximately +/−5, +/−15 and +/−25 from the dyad in their native fragments with their minor grooves facing inward toward the histone surface. Table 1C shows that the octamer sequences centered within these 10 bp insertions generally have high FVOs and confer high stability and positioning activity when tested experimentally in fragment 67. High frequency peaks in the occurrence profiles of the database sequences were also found at +/−15, +/−25 and +/−35 in most of these sequences (File S3) in agreement with their locations in the native fragments. Table 1D shows that the FVO values derived from the frequency profiles of the core tetramers, hexamers, and octamers within the sequences listed in Table 1 A–C are highly correlated with nucleosome stabilities and positioning activities. In contrast, the FVOs of central dinucleotides were not significantly correlated with these parameters (data not shown). These correlations indicate that sequence motifs known to be important for nucleosome positioning *in vitro* exhibit strong periodicities in genomic nucleosome libraries and that the intranucleosomal patterns of occurrence in these libraries is related to their activities in *in vitro* reconstitutions.

### Analysis of Tetranucleotides in *In Vivo* Libraries

A central question is whether the results obtained with nucleosomes reconstituted *in vitro* reflect the nucleosome sequence patterns found in cells. To address this question, tetranucleotide sequence profiles in 4 genome-wide *in vivo* libraries were characterized (File S2) and compared to those in the library generated by *in vitro* assembly. Three of the libraries were from yeast and one from *C. elegans* [8], [11], [12], [14] (Table 2). The *in vivo* libraries differ from the *in vitro* library in three aspects. First, there was no selection for high affinity octamer binding sequences, as there was for the *in vitro* sequences. Second, the *in vivo* nucleosomes positions are subject to nucleosome boundary effects where a nucleosome positioned by sequence can phase an adjacent nucleosome in a sequence-independent manner [8], [51]. Third, transcriptional-dependent processes have been shown to alter the positions of nucleosomes in yeast *in vivo* relative to the *in vitro* preferred positions, which are dictated by DNA sequence alone [13], [14], [22].

**Table 2:** General Characteristics of the Genome-Wide Nucleosomal DNA Sequence Libraries.

	Kaplan et al.	Kaplan et al.	Mavrich et al.	Weiner et al.	Valouev et al.
	2009	2009	2008	2009	2008
	*S. cerevisiae*	*S. cerevisiae*	*S. cerevisiae*	*S. cerevisiae*	*C. elegans*
	*In Vitro* R1	EtOH NOCL R1	H3H4	RPO21 0 min	SRX000425
**Coverage (reads/200 bp)**	79	55	35	39	71
**Average Tetra Periodicity +/−SD**	10.20+/−0.19	10.33+/−0.34	10.33+/−1.24	10.38+/−0.75	10.00+/−0.17
**Median Tetra Periodicity**	10.15	10.20	10.10	10.10	10.00
**Phase è Correl., r (** ***in vitro*** ** vs**. ***in vivo*** **)**	-	0.993	0.938	0.955	0.983
**Average Tetra FVO +/−SD**	0.068+/−0.034	0.026+/−0.013	0.025+/−0.013	0.015+/−0.008	0.025+/−0.010
**Average FVO Ratio (Tetra over Di)**	1.523	1.605	1.816	1.589	1.659

**Figure 6:**
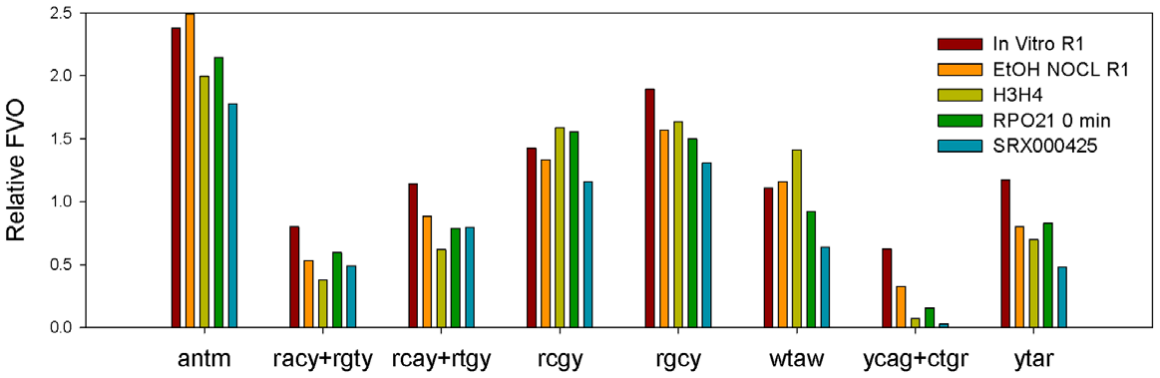
Periodicity analysis of tetranucleotide consensus sequences from libraries listed in **Table 2**. The FVO_10.2_ values for the tetranucleotide consensus sequences derived from the libraries listed in Table 2 were normalized by the average tetranucleotide FVO_10.2_ values from each library to determine the “relative FVO_10.2_ values”. These relative FVOs are grouped by consensus sequences in the vertical bar chart, and the different colors represent the different libraries, which are designated in the figure.

Table 2 displays global properties of all tetranucleotides sequences in the libraries while Figure 6 and Table S3 give characteristics of the 8 consensus sequences. The sequence features that reflect the patterns of occurrence and rotational orientations within nucleosomal DNA in the *in vitro* library are conserved in all *in vivo* libraries. The average periodicities of tetranucleotide sequences varied little among the libraries with an overall mean and median periodicity of 10.25 and 10.11 bp for those tetranucleotides that displayed significant FVOs. These periodicities are similar to those reported from the analysis of dinucleotides in chicken erythrocyte nucleosome DNA (10.15–10.26 bp) [35], and hydroxyl radical footprinting studies (10.18 bp) [52]. There was also a high correspondence between the phase angles in the *in vitro* and *in vivo* libraries as evidenced by the high correlation coefficients in Table 2 and in the correlation plots between the *in vitro* yeast data and the *in vivo* data from yeast and *C. elegans* (Figure S3). This correspondence can also be seen with all consensus tetranucleotides by the coincidence of peak frequency positions in the 8 consensus profiles in the *in vitro* and *in vivo* libraries (Table S3 and Figure S4).

The similarities between the *in vitro* and *in vivo* libraries extend beyond features that relate to rotational orientation of nucleosome DNA. The relative strengths of the tetranucleotides periodicities are also similar in the *in vitro* and *in vivo* libraries as revealed by the similarities in relative FVOs as seen in the Figure 6. These results suggest that the utilization of the consensus sequence tetranucleotides is similar *in vitro* and *in vivo* in both yeast and *C. elegans*. This is also seen from the qualitative similarities in occurrence profiles of tetranucleotides in the *in vitro* and *in vivo* libraries (Figures 2 and S4). These profiles also illustrate a consistent difference between the *in vitro* and all vivo libraries. The relative peak heights of all consensus sequences in the nucleosome periphery tended to be greater in the *in vivo* libraries as compared to the *in vitro* library. This difference was least pronounced with AnTm and most pronounced with YTAR.

The major difference between the *in vitro* and *in vivo* libraries was the strengths of the tetranucleotide periodicities, as quantified by FVOs. The FVOs of each tetranucleotide consensus sequence in each *in vivo* library, as well as the average tetranucleotide FVOs, are 3–4 fold lower than those displayed by the *in vitro* nucleosome sequences (Figures 2 & S3 and Table S4). It was also noted in previous studies that the strength of the AA/TT periodicity in *in vitro* libraries was greater than in *in vivo* libraries [12], [13]. Perhaps the most straightforward explanation for these results is that a smaller fraction of nucleosomes are positioned by DNA sequence *in vivo* [12], [13].

### Oligo A/T Tracts and Nucleosome Positioning and Stability

**Figure 7:**
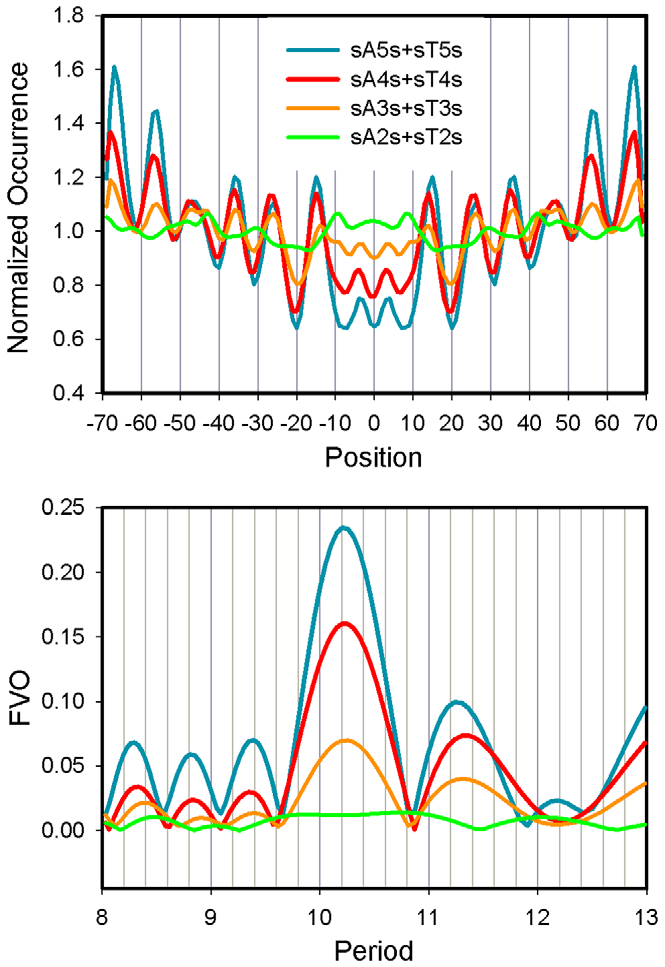
Periodicity analysis of isolated A/T sequences from the *in vitro* library. The occurrences of A_2_/T_2_, A_3_/T_3_, A_4_/T_4_, and A_5_/T_5_, isolated by C or G and normalized by their average frequencies, were computed for the top panel. Corresponding plots of FVO vs. period are displayed in the bottom panel. S = C or G.

Models attempting to explain nucleosome occupancy from nucleotide sequence are frequently based on 10 bp periodicities of dinucleotides, in particular AA/TT/TA. These studies most often quantify frequencies of AA and TT steps rather than individual di, tri, tetra, and penta-A and T-containing nucleotide motifs [7], [16], [29], [30]. A limitation to this approach is the uncertainty of the source of the signal since, for example, a single A_4_ tetranucleotide is counted as three AA dinucleotides. The importance of AA/TT dinucleotides in the *in vitro* yeast library was revaluated in Figure 7 by separating the signal qualities derived from isolated AA/TT dinucleotides and isolated A/T tracts of varying lengths. The sequence elements were isolated by G and C (S) rather than by T and A in order to eliminate tetranucleotides such as AATT, TTAA, and ATTA, which exhibit strong 10.2 bp periodicities (Table S1). The normalized occurrences are given in the top panel of the figure, and the corresponding FVOs as a function of period are graphed for these motifs in the bottom panel. Strong 10.2 bp periodicities are exhibited by the isolated oligonucleotide tracts, following the order A_5_/T_5_>A_4_/T_4_>A_3_/T_3_ while no significant patterns were detected with the isolated AA/TT dinucleotides.

**Figure 8:**
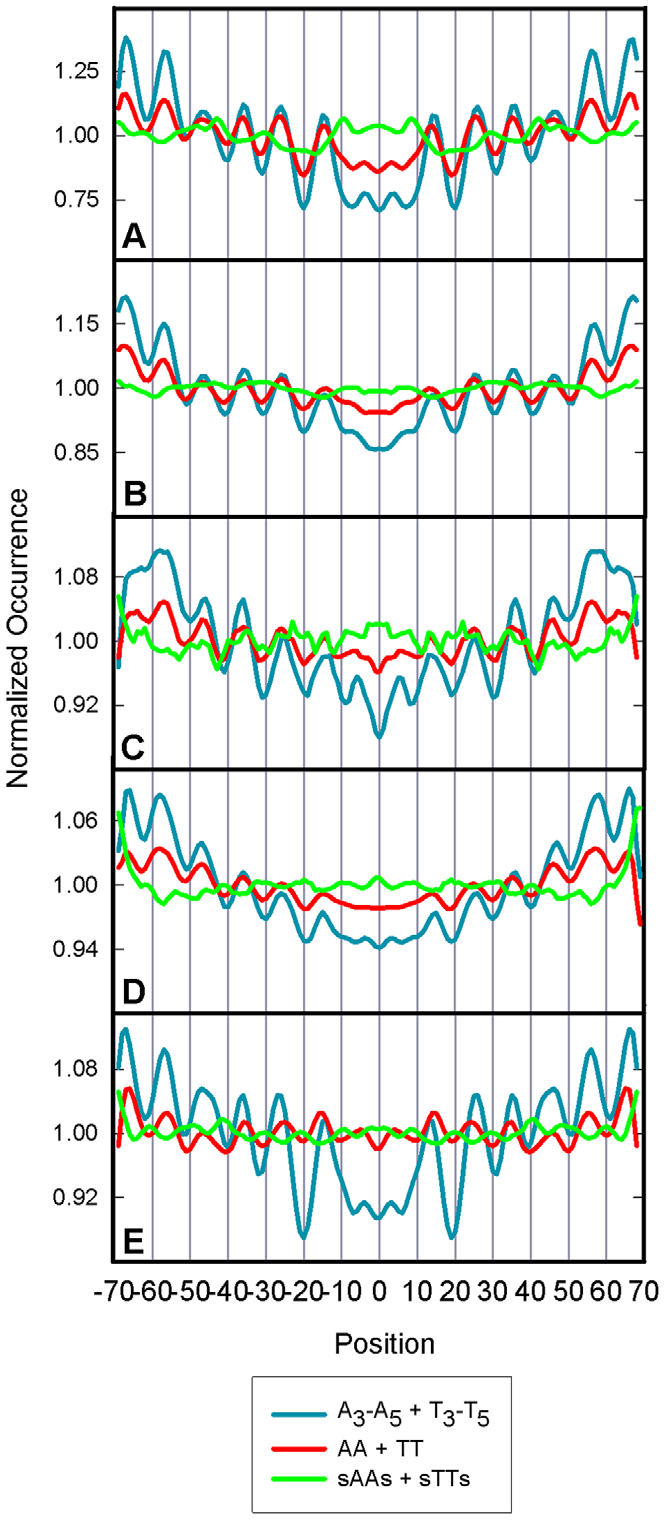
Occurrences of AA/TT dinucleotides, isolated AA/TT dinucleotides, and oligo A/T tracts. The occurrences of A/T sequence motifs along nucleosome DNA in the A) Kaplan et al. 2009 *in vitro* library, B) the Kaplan et al. 2009 EtOH non-crosslinked library [12], C) the Mavrich et al. 2008 library [8], D) the Weiner et al. 2009 library [14], and E) the Valouev et al. 2008 [11] (*C. elegans*) library were computed. The blue lines show the occurrences of oligo A_3_–A_5_/T_3_–T_5_ tracts, the red lines show the occurrences of all AA and TT dinucleotides, and the green lines show the occurrences of AA and TT dinucleotides isolated by C or G. All profiles displayed were normalized by their average frequencies and the FVOs, frequencies and periods are given in Table S4.

In order to provide additional evidence for the importance of oligo A/T tracts, the occurrences of isolated AA/TT, non-isolated AA/TT steps and oligo A_3_–A_5_/T_3_–T_5_, tracts were computed in the *in vitro* yeast library, the three *in vivo* yeast libraries and the library from *C. elegans* (Figure 8). The results revealed strong patterns for the oligo A_3_–A_5_/T_3_–T_5_, tracts, weaker patterns for non-isolated AA/TT steps and no significant patterns for the isolated AA/TT dinucleotides in each library. These occurrence profiles are reflected quantitatively in Table S4, which shows that the FVOs for oligo A_3_–A_5_/T_3_–T_5_, tracts were consistently ∼2- fold greater than those for the non-isolated AA/TT steps while the patterns for isolated AA/TTs are weak and not significant in all libraries. These results show that inclusion of the dinucleotides data in the total AA/TT step analysis detracts from the strength of the ∼10 bp relationship.

**Figure 9:**
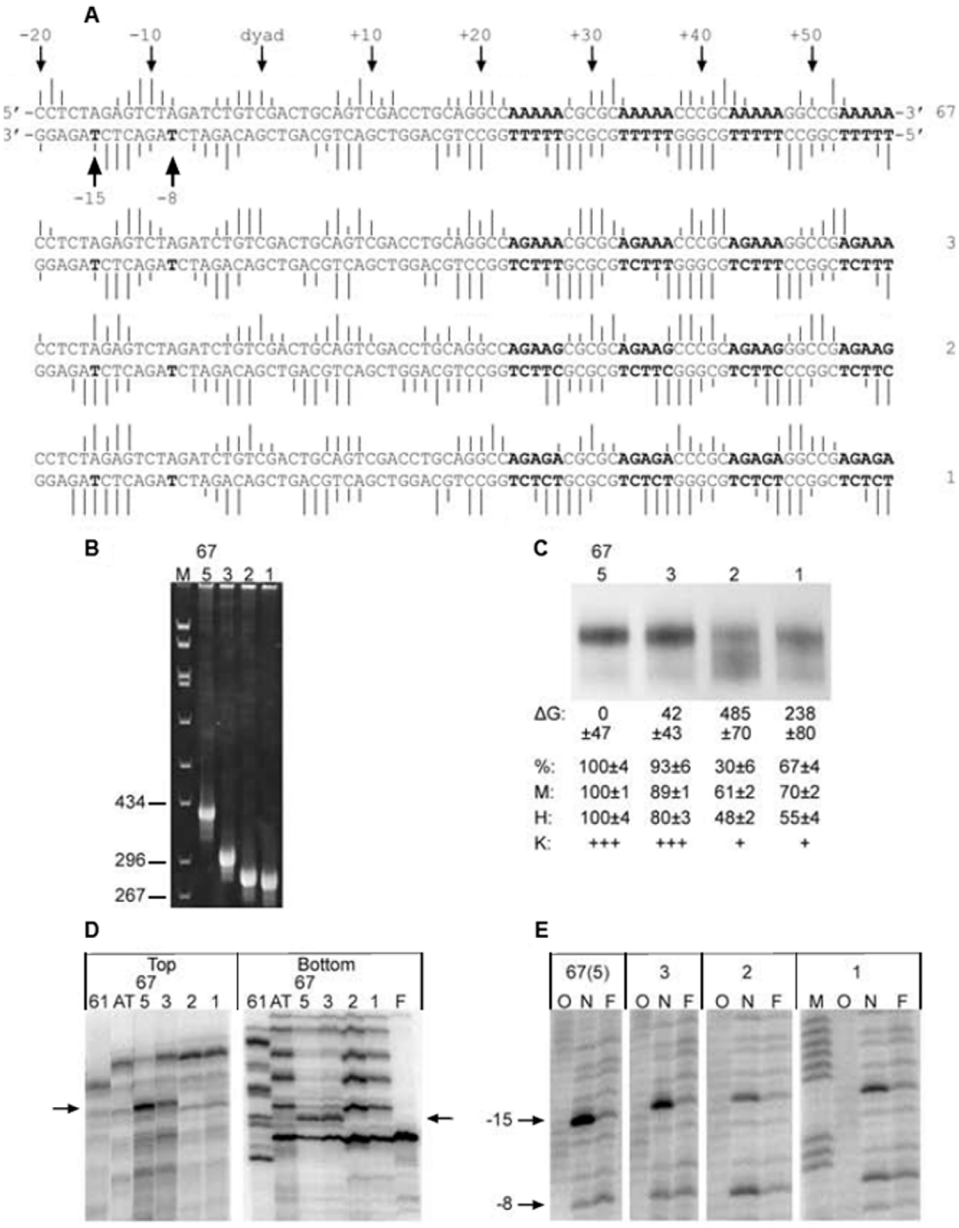
Effects of A-tract length on nucleosome positioning and stability. **A.** A portion of the sequence of Fragment 67 is shown at the top [36], [37], [54]. The four A5 tracts that are downstream of the nucleosome dyad are indicted in bold face type. The upward facing arrow on the far left indicates the KMnO_4_ hypersensitive T residue at −15 on the bottom strand. The sequences under Fragment 67 indicated in bold are the altered A-tracts, which corresponded to A_3_ (AGAAA), A_2_ (AGAAG), and A_1_ (AGAGA). Hydroxyl radical cleavage efficiency is indicated at each base by vertical lines. **B.** Fragments were separated on a 9% native PA-gel at 5°C in order to study DNA bending. M is a marker. **C.** Fragments were reconstituted into nucleosomes at 25°C using the histone exchange procedure and energies of reconstitutions were determined as described previously using chicken DNA as competitor. Translational positioning was determined on native PA gels and a sample gel is shown in the figure. Over 95% each fragment was assembled into nucleosomes and only the nucleosome region of the gel is shown in the figure. The % of radioactivity in the top-positioning band is given below the figure as are the results of restriction nuclease accessibility measurements for positioning activity (M, Msp 1; H, Hae III). **D.** Fragments were end labeled either on the top or bottom strands and assembled into nucleosomes. Reconstituted fragments were then digested with Exo III for 5 minutes. The arrows indicate the nucleosome boundary of Fragments A_5_ (67) and A_3_. Lane F corresponds to free DNA of Fragment 67. Fragments 61 and AT were used as negative controls. **E.** Sequencing gel showing the KMnO_4_ reactivity in nucleosome (N) or free DNA (F) of fragments A_5_, A_3_, A_2_ and A_1_. Arrows indicate the positions, relative to the dyad, of the KMnO_4_ hypersensitive sites. Lane O are products of hydroxyl radical cleavage reactions. Relative intensities of the KMnO_4_ sites at −15 are given in **C**.

Several studies have suggested that oligo A/T tracts in a 10 bp period can facilitate nucleosome formation but the sequence features that are responsible for this effect have not been clearly defined [31]–[34]. Since the studies in Figures 7 and 8 show that isolated AA/TT dinucleotides are not periodic in genomic nucleosome sequences, it was of interest to examine the effects of A-tract length on promoting nucleosome assembly. The synthetic nucleosome positioning sequence Fragment 67 was used to address this question (Figure 9). The fragment was modeled after natural nucleosome positioning sequences and contains two regions of curvature that reside on opposite sides of the dyad [53], [54]. As noted above, the fragment also contains a single KMnO4 hypersensitive site that is located at a TA step at −15 bp from the dyad, which is required for high nucleosomes stability and unique positioning [36], [37]. The four A tracts depicted in the figure alternate with GC rich segments, and their minor grooves face the histone surface. The region containing these tracts is responsible for establishing the rotational orientation of the entire fragment [54]. In order to characterize the effects of A-tract length on nucleosome properties, the four A5 (AAAAA) tracts in Fragment 67 were replaced by A3 (AGAAA), A2 (AGAAG) and A1 (AGAGA) sequences. Electrophoretic analysis of the four 223 bp fragments on the PA bending gel in Figure 9B revealed that the electrophoretic anomaly displayed by the wild type A5 (67) fragment was reduced by approximately 50% and 80% upon conversion to A3 and A2, respectively. The electrophoretic mobility of the A2 fragment is essentially the same as fragment A1, illustrating the importance of at least 3 continuous As in generating electrophoretic anomaly, in agreement with previous reports [55].

The four fragments were reconstituted into nucleosomes at 25°C and 37°C using the histone exchange method, and the nucleosomes were analyzed in order to determine if they could promote high nucleosome stability and translational positioning activity (Figure 9C). Translational positioning activity was first analyzed by native PAGE analysis where the slow migrating nucleosomes are located on the positioning sequence in the center of the fragment. The nonpositioned nucleosomes assemble at multiple sites along the sequence, and consequently, most migrate faster than the centrally positioned nucleosomes. Representative samples of these native gels are shown in the figure. Positioning activity was also monitored by restriction endonuclease accessibilities using Hae III and Msp I as detailed previously [36], [37]. The data are summarized below the gel as the means (+/−S.E.M.) from at least 4 independent experiments. The results demonstrated that there was a modest decline in stability and positioning activity in the A3 construct relative to the wild type A5 (67) sequence but a dramatic decrease at both temperatures in the A2 and A1 fragments. The high translational positioning activity associated with the A5 and A3 fragments was also evident from exonuclease III digestion patterns in Figure 9D. Digests of nucleosomes reconstituted onto Fragments A5 (67) and A3 revealed major persistent pauses that mapped to the borders of the positioned nucleosome as reported previously for Fragment 67 [36], [37]. In contrast, the A2 and A1 nucleosomes displayed a ladder pattern, indicative of multiple nucleosome positions on these fragments. The ladder pattern is essentially identical to that seen with the negative controls, Fragments AT and 61. Fragment AT has an AT step in place of the TA step at the −15 site of Fragment 67 while Fragment 61 is a 6 bp deletion of Fragment 67.

The rotational orientation of the TA step at the −15 site is important for the high nucleosome positioning activity and stability of Fragment 67 since reductions in these functions were seen when the TA step was translocated by as little as 1 bp in either direction [37]. This observation, and the observation that these 4 A5 tracts shown in the figure likely dictate the rotational orientation of the entire fragment [54], provide a plausible mechanism by which the A5 tracts control positioning since these tracts should dictate the rotational orientation of the TA step. To investigate this possibility further, reconstituted nucleosomes were subjected to hydroxyl radical cleavage analysis (Figure 9E), and the relative strengths of the hydroxyl radical cutting sites are indicated by the vertical lines in Figure 9a. In Fragments A5 (67) and A3 , the minor grooves of the downstream A tracts face the histone surface, as does the minor groove of the upstream TA step at −15bp. However, the rotational orientation displayed by Fragments A2 and A1 was shifted by 2–3 bp, producing an altered rotational orientation of the TA step at −15 from an inward to a more outward facing position in relation to the histone octamer (Fig 9a). This altered rotational orientation likely plays a role in the near loss of the KMnO4 hypersensitivity of the reactive T at the TA step in fragments A2 and A1 (Figure 8E), as well as the low positioning activities of these fragments (Figure 9C,D). These results point to the importance of oligo A tracts in the establishment of rotational orientation and illustrate how rotational orientation can be directly linked to translational positioning. The phased A-tracts may also contribute to the stability of Fragment 67 by an effect independent of the action on translational positioning since curved sequences without translational positioning signals are preferentially packaged into nucleosomes [32], [33].

## Discussion

### Tetranucleotides vs. Dinucleotides

Perhaps the most distinguishing sequence characteristic of positioned nucleosomes is the periodic occurrences of certain dinucleotides, and this feature forms the basis of many models that have been used for predicting nucleosome occupancy from nucleotide sequence [7], [16], [28], [29], [35]. Some of the more recent models also incorporate non-periodic and position-independent sequence characteristics including oligonucleotides, G+C content, and long A tracts as nucleosome exclusion elements [11], [12], [29], [56]–[60]. In this report, we have taken an approach for describing positioned nucleosomes, which exploits sequence information derived from the periodic occurrences of the 256 tetranucleotides in nucleosome DNA. The approach entailed the identification and analysis of 24 unique tetranucleotides that were defined by 8 consensus sequences (Figures 1 and 2). The periodicities of these 24 tetranucleotides are responsible for most of the strength of the tetranucleotide periodicity displayed by the entire *in vitro* library, and consequently the 8 consensus sequences are the major source of the periodic signals in positioned nucleosomes. In addition, the signal strength displayed by the consensus tetranucleotides increased dramatically with increasing sequence reads, which is expected of sequence elements that are responsible for nucleosome positioning and/or histone binding affinities. The consensus tetranucleotides are also responsible for essentially all of dinucleotide periodicities displayed by the library as seen in Figures 3 and S2, which points to the fundamental importance of these tetranucleotides, in contrast to dinucleotides, as distinguishing features of positioned nucleosomes.

The analysis of A-tract length on nucleosome positioning also provided strong evidence that oligonucleotide-length sequences rather than dinucleotides give a more accurate and complete description of sequence features that are involved in nucleosome positioning. The studies in Figure 9 demonstrated that an A_3_-containing DNA fragment arranged in a 10 bp period displayed approximately half the nucleosome positioning activity and electrophoretic anomaly when compared to an A_5_ fragment, while A_2_ and A_1_-containing fragments displayed near normal gel mobility, low affinity for the histone octamer and failed to position nucleosomes at single translational sites *in vitro*. These results are in total agreement with the studies in Figures 7, 8 and Table S4, which show that the source of the periodic signal seen in the analysis of all AA/TT steps is due to oligo A3–A5/T3–T5 tracts, and that isolated AA/TT dinucleotides are not periodic in genomic nucleosome sequences from yeast and *C. elegans*. In fact, the present results clearly show that inclusion of isolated AA/TT dinucleotides detracts from the strength of periodicities when all AA/TT steps are computed in both *in vitro* and *in vivo* libraries, which raises the question as to whether isolated AA/TT dinucleotides should even be included in predictive models for nucleosome occupancy.

The nucleotides that flank central dinucleotides in a tetranucleotide can have profound influence on the properties of the tetramer [42]–[47]. For example, each AA dinucleotide embedded in an oligo A-tract of 3 bp or longer has a highly unusual structure that confers to the tract enhanced stiffness and resistance to bending forces. In contrast, the structure and properties of AA dinucleotides flanked by G or C are characteristic of normal B-DNA [26], [48], [55], [61], [62]. Similarly, the ability of the TA step to facilitate nucleosome assembly and positioning in regions of high curvature demand in the nucleosome is highly dependent on the nature of the TA flaking bases [36], [37, Figure 5 and Table 1]. Flanking bases of central dinucleotides also often play a significant role in dictating the phase angle of a tetranucleotide and consequently whether the minor groove of the tetranucleotide faces toward or away from the histone surface. This effect was seen with each of the 10 unique central dinucleotides as detailed in File S2. For example, the dinucleotide CA/TG displays a weak 10.2 bp base periodicity; however, the tetranucleotide analysis in Figure 2 resolved two distinct patterns for CA/TG (RCAY+RTGY and YCAG+CTGR), which display opposite rotational orientations causing a cancellation of signal strength in the dinucleotide profile. TA and TG/CA steps are the most intrinsically variable and hence most flexible of the ten unique dinucleotides in terms of roll, twist and slide. The TG/CA step is also the most variable in terms of bending into the minor and major grooves as revealed by analysis of crystal structures of oligonucleotides and *in vitro* studies with positioned nucleosomes [42]–[44], [49], [63]. The results presented in this study suggest that this distinction is related to the TG/CA flanking bases in the nucleosome.

Studies with natural and synthetic nucleosome positioning sequences have suggested that the major determinants for translational positioning are located in the central regions of nucleosomal DNA [1]. The center turns of the nucleosomal DNA at positions 0 to +/−30 are tightly associated with the H3/H4 tetramer, and the sharpest bends in the nucleosome occur in this region at +/−15 bp from the dyad [64]–[67]. Previous experimental studies with synthetic fragments and the 5S rDNA sequence from sea urchin have shown that the nucleosome positioning sequences TTAA and CTAG with the consensus YTAR are located at sites of maximal curvature in the nucleosome at positions +/−5, +/−15 +/−25 and +/−35 bp from the dyad [36]–[38]. There is a preference for TA containing motifs over TG motifs in these central turns as seen in nucleosome occupancy profiles, which is consistent with the observation that nucleosome positioning activity followed the order CTAG>CTGG>CNNG when these sequences were placed at the −15 bp region in a synthetic nucleosome positioning sequence [37]. Richmond and Davey [68] demonstrated that DNA kinking occurred at TG steps at positions −+35 −/+45 and +/−55. The inward facing CTGR+YCAG that overlap with these regions is consistent with this view. The sequences RCAY+RTGY, RACY+RGTY, RCGY and RGCY that are centered 5 bp away from the YTAR and YCAG+CTGR elements could play a role in facilitating the DNA bending into the major groove in the central turns of nucleosomal DNA (Figures 2, S4). All of these outward-facing sequences have relatively wide minor grooves (6.8–7.7 A), which favor the deflection of the helical axis toward the histone surface. This arrangement was also observed in the central region of the nucleosome positioning sequences analyzed in Figure 4, which contain an unusually high density of both inward and outward facing consensus tetranucleotodes. These results seem consistent with the mini-kink model for DNA bending in the nucleosome, where DNA sharply bends into the minor and major groove at 5 bp intervals by a mechanism that involves lateral slide displacements [69].

### *In Vitro* vs. *In Vivo* Libraries

Yeast genome -wide studies have established that most nucleosomes are positioned at the same chromosome location in the majority of the cells in the population [4], [5], [6], [8], [12]–[14]. However, the fraction of nucleosomes that are positioned by DNA sequence in the cell remains an open question. While it is clear that long dA∶dT tracts in NFR regions are important factors in promoting nucleosome exclusion both *in vitro* and *in vivo* [26], it remains uncertain as to the prevalence of nucleosome favoring sequences in the genome. One common approach used to address this question has entailed the comparison of *in vivo* and *in vitro* nucleosome occupancy maps [12]–[14]. Although these studies have often led to controversial results and interpretations, most of the recent analyses have suggested that the fraction of nucleosomes positioned by sequence *in vivo* is small, and that epigenetic factors play more influential roles in nucleosome organization. Recent observations have also shown that depletion of a chromatin remodeling factor [22] and RNA polymerase [14] resulted in nucleosome repositioning to a state that is more similar to the positions dictated by DNA sequence as detected by *in vitro* reconstitution. These results are consistent with the emerging view that the ground state of nucleosome organization is dictated by DNA sequence and that epigenetic factors are superimposed on this state for the final organization of nucleosomes in the cell.

These considerations raise questions concerning the results of the studies described in this report. The results of this study revealed that the nucleotide sequence patterns of nucleosome positioned *in vivo* are strikingly similar to those assembled *in vitro* from purified components. These similarities include rotational orientations and relative FVOs of tetranucleotides, tetranucleotide periodicities, and the profiles of occurrence of the consensus tetranucleotides, which are likely to play important roles in nucleosome positioning (Figures 2, 6, S2, S4). In addition, an analysis of the yeast *in vivo* library by the procedures described in Figure 3 revealed that the periodicity of dinucleotides was due primarily to the tetranucleotides that comprise the 8 consensus sequences as was seen with the *in vitro* library (data not shown).

The major difference between the *in vitro* and *in vivo* libraries was the strengths of the tetranucleotide periodicities, as quantified by FVOs. The FVOs of the tetranucleotide consensuses sequences in each *in vivo* library, as well as the average tetranucleotide FVOs, are 3–4 fold lower than those displayed by the *in vitro* nucleosome sequences (Table 2 and Table S3). These results imply that the frequency of positioning determinants on a sequence basis is less in the *in vivo* datasets or, more likely, that a relatively large fraction of the sequences in the *in vivo* libraries lack sequence information for DNA directed nucleosome positioning. It follows that the small subset of sequences in the *in vivo* libraries that contain positioning signals may be derived from those nucleosomes that have not been subjected to repositioning by transcription, epigenetic mechanisms or chromatin boundary effects. These residual nucleosomes might be expected to represent a relatively small fraction of the yeast genomic sequences since at least half of the yeast genome is transcribed at least once during the cell cycle. These results should not be taken to imply that DNA sequence-directed nucleosome positioning is not of biological relevance since nucleosomes positioned by DNA sequence may be important for the initial repositioning processes. For example, nucleosomes positioned by DNA sequence can control the initial direction of translocation, translocation distance, as well as the new positions adopted by nucleosomes in response to chromatin remodeling machines [70].

Trifonov and Sussman [28] identified 10 bp sinusoidal patterns of AA/TT dinucleotide sequence preference along eukaryotic DNA nearly 30 years ago and suggested that these patterns facilitate the packaging of DNA into the nucleosome. This interpretation has been used extensively as evidence for the relevance of periodic sequence patterns in genome-wide nucleosome libraries. A more direct way for assessing biological significance of these patterns is based on the results in Figures 5, 6 and Table 1, which show that sequence features known to be important for nucleosome positioning *in vitro* were enriched in the genome-wide libraries, and that their rotational orientations and distributions along nucleosome DNA were correlated with their activities in *in vitro* reconstitutions reactions. Likewise, the effects of A-tract length on nucleosome stability and positioning activity as revealed by in *in vitro* assembly was highly correlated with the strength of the periodic patterns of these A sites in the *in vitro* and *in vivo* sequence libraries (Figures 7, 8, 9 and Table S4). The high correspondence between the strengths of the patterns in genome-wide libraries and positioning properties *in vitro* points to a basic strategy that could be used for development of novel predictive models for identifying nucleosome positions from nucleotide sequence and for evaluating sequence heterogeneity in nucleosome libraries in a meaningful fashion. Such a strategy could be used for fractionating nucleosome libraries into sequence subsets with different positioning determinants, and for assessing the number, arrangement, and linkage of positioning motifs in specific subsets of nucleosomal DNA sequences.

## Materials and Methods

The yeast nucleosomal DNA sequence libraries analyzed in this study were derived from nucleosome occupancy experiments performed by Mavrich et al. 2008 [8], Weiner et al. 2009 [14], and Kaplan et al. 2009 [12]. The Mavrich et al. 2008 data (yeast-H3H4-reads.txt) were downloaded from the Penn State Genome Cartography Project website: ftp://ftp.sysbio.bx.psu.edu/h3h4/. The Weiner et al. 2009 data were obtained from the Gene Expression Omnibus under accession number GSE18530 (GSM461564 -RPO21 0 min). The Kaplan et al. 2009 data were acquired from GEO under accession number GSE13622.

The *in vitro* replicate 1 library (GSM351491) and the YPEtOH non-crosslinked *in vivo* library (GSM351494) from the Kaplan et al. 2009 data were used in this study. The information from these sources provided the yeast genome coordinates as well as the number of reads for each procured sequence. The Kaplan and Weiner data provided the 5′ ends of the reads with directionality while the Mavrich data provided the nucleosome midpoints. These coordinates were used to extract nucleosomal DNA sequences from the May 2006 build of the Saccharomyces Genome Database. All sequences were made to be 147 bp in length, and the reverse compliments of these sequences were also analyzed. When computing the frequency profiles of a given motif, each nucleosomal DNA sequence was weighted by its corresponding number of reads (Figure S5). So, if a certain sequence in one of these libraries had four reads, a given motif at any given position along the nucleosomal DNA would be counted four times instead of just once.

Five of the 13 *in vivo* libraries prepared by Kaplan displayed similar tetranucleotide profiles to the YPEtOH non-crosslinked library (YPEtOH crosslinked replicates 1 and 2, YPGal crosslinked replicate 1, and YPGal non-crosslinked replicates 1 and 2). The YPEtOH non-crosslinked replicate 1 library was chosen at random from this group of six for presentation in this report. The remaining seven *in vivo* libraries were out of phase by 5 bp as judged by the phase angles of AAAA and by a variety of other criteria. This may have resulted from slight over trimming or under trimming by MNase, but other explanations cannot be excluded. These libraries were rendered in phase by adjusting the phase angles of AAAA to +/−180 degrees through shifting the sequences 5 bp. When these adjusted libraries and the six unadjusted libraries were analyzed as a group of 13, the tetranucleotides profiles were nearly indistinguishable from the YPEtOH non-crosslinked library (data not shown).

The *C. elegans* nucleosome data were derived from studies conducted by Valouev et al. 2008 [11] and acquired from the Short Read Archive at NCBI under accession number SRA001023 (SRX000425). These short reads were mapped using the Bowtie software and pre-built indexes for the most recent assembly of the *C. elegans* genome [71]. The default two-mismatch threshold was applied along with “–m 1” reporting mode to ensure that only unique, confidently mapped reads were utilized. The color space option was used for these SOLiD reads. To report the 5′ end coordinates of the reverse reads instead of the 3′ ends, the following post-processing code was added to the command line:
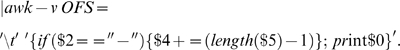
With these inputs, 33% of ∼110 million *C. elegans* reads were reported. All nucleosomal DNA sequences from *C. elegans* were analyzed with their reverse complements and were made to be 147 bp in length.

### Generation of the Modified *In Vitro* Libraries

The “consensus only” and “non-consensus” libraries were derived from the Kaplan et al. 2009 *in vitro* replicate 1 library. Nucleotides within the sequences of the “consensus only” library were replaced with an “X” unless they occurred within a consensus tetranucleotide. The consensus tetranucleotides were allowed to overlap each other. For example, no part of the sequence, ACGTGT, would be converted to an “X” because it is an overlap of the consensus tetranucleotides, RCGY and RCAY+RTGY. Because the consensus tetranucleotides were allowed to overlap, non-consensus tetranucleotides could also be counted in this library (such as CGTG, which is contained within the example sequence ACGTGT). However, the occurrences of non-consensus tetranucleotides in this library were considerably less than the occurrences of consensus tetranucleotides as expected (data not shown). Replacing nucleotides with “X's” in the “consensus only” library allowed one to count only tetranucleotides and dinucleotides that occurred within isolated or overlapping consensus tetranucleotides. ∼51% of the nucleotides in the “consensus only” library were replaced with an “X.” The “no consensus” library was the exact opposite of the “consensus only” library as all nucleotides within isolated and overlapping consensus tetranucleotides were replaced with an “X.” In the “no consensus” library, the 40 (or 24 unique) consensus tetranucleotides had zero occurrences. ∼49% of the nucleotides in the “no consensus” library were replaced with an “X.”

### Counting Occurrences of Motifs with Different Lengths

In order to analyze the periodicities of motifs of various lengths, a standard method for counting their occurrences was developed. The following formula was used to determine what position a particular motif would be counted:
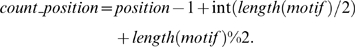
Therefore, the dinucleotide centers of even-length motifs are counted at the same position. For example, if a TA step was counted at −15 from the dyad and its 5′ and 3′ flanking bases were C and G, respectively, the corresponding tetranucleotide CTAG would also be counted at −15. Additionally, by this method, the centers of all odd-length motifs are counted at the same position. All frequency profiles displayed were generated after being subjected to a three-bond averaging procedure. To calculate the normalized occurrences for a frequency profile, the number of occurrences at each position along the nucleosomal DNA was divided by the average number of occurrences from all positions along the nucleosomal DNA.

### Fourier-Transform Analysis

To measure the periodicities of the motif occurrences, Fourier-transform analysis was carried out on the raw frequency data as implemented previously [35], [72]. The frequency-domain complex coefficients (*C_h_* = *A_h_*+*iB_h_*) are given by
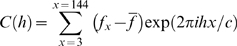
where 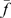 is the average frequency, 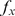 is the frequency at position x, and *c*/*h* is the period. To generate the Fourier-transform spectra, the amplitude |*C_h_*| was calculated over a range of periods by incrementing *h* from 250 to 150, corresponding to periods of 8 to ∼13.5 bp, respectively, as *c* = 2000. The values of *c* and *h* were selected in order to control the bp intervals in the Fourier-transform spectra.

### Evaluation of Motif Periodicities

The fractional variation of occurrence (FVO) was used to determine and compare the strengths of the periodicities among the various motifs [35]. The FVOs were calculated from Fourier-transform (FT) spectra at either the maximal amplitude (FVO_MAX_), or a 10.2 bp period (FVO_10.2_). A period of 10.2 bp was selected since the majority of tetranucleotides' maximum amplitude periodicities were near 10.2 bp (Figure S6). The equation used to determine the FVO for a 10.2 bp periodicity is given below
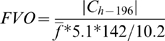
where |*C_h = 196_*| represents the amplitude in the FT spectrum at period of 10.2 bp and 142/10.2 represents the number of periods between positions x = 3 and 144.

The periodicities were also assessed by a technique that involved calculating areas under the Fourier-transform spectra. The value, %FTS_10.2_, was developed and is characterized by the percent area under the Fourier-transform spectra (%FTS) from 9.8 to 10.6 bp over the area under the entire FT spectra (8 to ∼13.5 bp). These %FTS_10.2_ values for many motifs were compared to their corresponding FT spectra, and this relationship was used to establish the significance of a given motif's 10.2 bp periodicity.

### Computation of Phase Angles

The phase angles were based on 10.2 bp periodicities and were calculated using a reference point at position 2 (−72 from the dyad) from using the equation 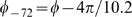 where 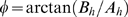 and *h* = 196 [35]. Therefore, a motif will have a phase angle of 0° if its frequency maxima are located at positions −72, −61.8, −51.6, −41.4, −31.2, −21, −10.8, −0.6, 9.6, 19.8, 30, 40.2, 50.4, 60.6, and 70.8 relative to the dyad.

### Table 2 Calculations

For determining the average and median tetranucleotide periods, tetranucleotide periods in the individual libraries were excluded if their corresponding FVO_MAX_ values were less than one-half SD below the mean FVO_MAX_ value of all tetranucleotides. For determining the correlation coefficients of the phase angles between the *in vitro* and *in vivo* libraries, tetranucleotide phase angles in the individual libraries were excluded if their corresponding FVO_10.2_ values were less than one-half SD below the mean FVO_10.2_ value of all tetranucleotides.

### Experimental Studies in Figure 8

All procedures used in these studies have been described previously [36], [37], [54]. The three derivative fragments were produced by insertion of synthetic oligonucleotide duplexes into the Psha I and Hind III sites of fragment 67. The calculated R_L_ (apparent length/real length) of the fragments on the PA gel shown in Figure 8B were 1.8, 1.4, 1.2, and 1.2 for fragments A_5_ (67), A_3_, A_2_ and A_1_, respectively. The residual electrophoretic retardation seen in Fragments A_2_ and A_1_ is due to the four dyad upstream tracts, which are common to all fragments. Reconstitution procedures were also carried at 37°C, and the energies of reconstitutions were −42+/−40, 202+/−202, 879+/−111, and 721+/−75 cal/mol for A_5_ A_3_ A_2_ and A_1_ fragments, respectively. The corresponding percentages of radioactivities in the positioning bands on native gels were 97, 80, 28 and 38. Note that the −8 site in A and E has the same core sequence (CTAG) that is found at −15. The T at −8bp on the bottom strand in Fragments A_2_ and A_1_ becomes slightly more sensitive to permanganate because of the altered rotational orientation in these fragments.

## Supporting Information

Figure S1 Elimination of tetranucleotides with weak periodicities. Only tetranucleotides that displayed significant ∼10 bp periodicities in the Kaplan et al. 2009 *in vitro* library were used for the development of the tetranucleotide consensus sequences. This was determined from the %FTS_10.2_ values, which represent the percent area under the Fourier-transform spectra (%FTS) from 9.8 to 10.6 bp over the area under the entire FT spectra of 8 to ∼13.5 bp. Tetranucleotides were included if they had %FTS_10.2_ values greater than 19% and FVO_10.2_ values greater than one-half standard deviation below the mean FVO_10.2_. Examples of tetranucleotides that display strong, borderline and weak FT spectra are shown in the figure. About 30% of the tetranucleotides were eliminated because of low %FTS_10.2_ scores. A high correlation was exhibited between the FVO_10.2_ and %FTS_10.2_ values (r = 0.79). Consequently, the cutoff point of a 19% FTS_10.2_ value eliminated nearly all of the tetranucleotides with FVO_10.2_ values that were less than 0.051, which was one-half standard deviation below the mean FVO_10.2_. Only a few tetranucleotides with %FTS_10.2_ values less than 19% had FVO_10.2_ values greater than 0.051. An exception was made for the inclusion of CTGA/TCAG into Figure 1 because its frequency profile was periodic in the central region (File S1).
(0.27 MB TIF)

Figure S2 Contribution of the consensus tetranucleotides to the periodicities of the 10 unique dinucleotides. The Kaplan et al. 2009 *in vitro* replicate 1 library [12] was modified as described in the Methods Section in two different ways in order to evaluate the significance of the consensus tetranucleotides. Panels A, B, and C present graphs of FVO versus period for the 10 unique tetranucleotides for the Unmodified, Consensus Only, and No Consensus libraries, respectively, for sequences with greater than six reads.
(0.20 MB TIF)

Figure S3 Phase angle plot (*C. elegans* and EtOH NOCL R1 vs. *In Vitro* R1). The frequency profiles of tetranucleotides from all the sequences in the *in vitro* replicate 1, the EtOH non-crosslinked replicate 1, and the *C. elegans* nucleosomal DNA sequence libraries were examined in order to calculate the phase angle for each tetranucleotide. The phase angles of the two *in vivo* libraries, EtOH non-crosslinked replicate 1 and *C. elegans*, were plotted against the *in vitro* replicate 1 library, yielding Pearson correlation coefficients of 0.99 and 0.98, respectively. For this phase angle correlation, approximately 30% of the phase angles from each of these three libraries were omitted because their corresponding FVO_10.2_ values were less than one-half standard deviation below the mean.
(0.26 MB TIF)

Figure S4 Tetranucleotide consensus sequence profiles of select *in vivo* libraries. The frequency profiles of the tetranucleotide consensus sequences are displayed for the Kaplan et al. 2009 ethanol non-crosslinked replicate 1 and the Valouev et al. 2008 (*C. elegans*) nucleosome occupancy experiments.
(2.24 MB TIF)

Figure S5 FVO analysis of motifs with different lengths. For the *In Vitro* Replicate 1 library, the FVO_10.2_ values were determined for nucleotide sequence motifs that ranged in length from 1–6 nucleotides in order to study the relationship between sequence length and enrichment of 10.2 bp periodic sequences in sub-libraries with increasing numbers of reads. The mean FVO_10.2_ values for each sequence length are plotted against the number of reads in the sub-libraries. The SD for each point ranged from +/−40–60% of the means. The results show that the mean FVO_10.2_ for each sequence length increased as function of the number of reads, and that the longer sequences increased to a greater extent than the shorter ones. Due to this observation, all nucleosomal DNA sequences were weighted by the number of reads in this study. Randomized subsets of the total library did not increase the FVO_10.2_ values, which indicate that the smaller number of sequences in the higher-read libraries are not causing the increases in the FVO_10.2_ values (data not shown).
(0.11 MB TIF)

Figure S6 Tetranucleotide periodicities in the *in vitro* library. From the Fourier-transform spectra of each tetranucleotide, the maximum amplitude period over a range of 8 to ∼13.5 bp was determined for each tetranucleotide in the *in vitro* library. A histogram with bin widths of 0.1 bp over a range of 9.65 bp to 10.75 bp is displayed below and shows that the majority of the tetranucleotide maximum amplitude periods are near 10.2 bp.
(0.08 MB TIF)

Table S1 Tetranucleotide characterization in the Kaplan et al. 2009 *in vitro* library. The 256 tetranucleotides are arranged in the table according to the 10 unique dinucleotide steps located in the center of the tetranucleotides. Reverse complements are also paired. Fourier-transform analysis of the tetranucleotide frequency data was carried out for each tetranucleotide in the Kaplan et al. 2009 *in vitro* library in order to calculate the maximum amplitude periodicities, the FVO_MAX_ and FVO_10.2_ values, the %FTS_10.2_ values, and the phase angles. Additionally, each tetranucleotide FVO_10.2_ was normalized by the average FVO_10.2_ of all tetranucleotides.
(0.06 MB XLS)

Table S2 Dinucleotide characterization in the Kaplan et al. 2009 *in vitro* library. In order to compare the tetranucleotides with their center dinucleotides, a table was constructed for the 16 dinucleotides containing the maximum amplitude periodicities, the FVO_MAX_ and FVO_10.2_ values, the %FTS_10.2_ values, and the phase angles. The normalized FVO_10.2_ values for each dinucleotide are also included.
(0.19 MB XLS)

Table S3 Analysis of the tetranucleotide consensus sequences for the nucleosome libraries. The maximum amplitude periodicities, the FVO_MAX_ and FVO_10.2_ values, and the phase angles are displayed below for the tetranucleotide consensus sequences in the four *in vivo* libraries as well as the *in vitro* library listed in Table 2. It is important to note that due to the fact that the reverse complement pairs of dinucleotides and tetranucleotides possess opposite phase angles, the sum of the frequency profiles of reverse complement pairs will always possess phase angles of 0 or +/−180 degrees. If dinucleotides or tetranucleotides within a reverse complement pair are far from 0 or +/−180 degrees, the corresponding FVO of the reverse complement pair will decrease relative to the FVOs of the single components. On the other hand, if dinucleotides or tetranucleotides within a reverse complement pair are close to 0 or +/−180 degrees, the corresponding FVO of the reverse complement pair will reflect the FVOs of the single components. If a perfect reference point had been utilized in calculating the phase angles, the table would display 0's and +/−180's instead of −177.5's and 2.5's.
(0.06 MB DOC)

Table S4 Analysis of the periodicities for profiles in figure 8. The average frequencies, maximum amplitude periodicities, and FVO_10.2_ values are displayed for select motifs derived from the A) Kaplan et al. 2009 *in vitro* library, B) the Kaplan et al. 2009 EtOH non-crosslinked library, C) the Mavrich et al. 2008 library, D) the Weiner et al. 2009 library, and E) the Valouev et al. 2008 *C. elegans* library. NS = Not Significant.
(0.04 MB DOC)

File S1 Frequency profiles of tetranucleotides in figure 1. The frequency profiles of the tetranucleotides derived from the Kaplan et al. 2009 *in vitro* library that are shown in Figure 1 are displayed within the file.
(1.58 MB XLS)

File S2 Tetranucleotide & dinucleotide characterization of *in vivo* libraries. Separate tables identical to Table S1 and Table S2 were generated for each library listed in Table 2 and are located in File S2. In these tables, the 256 tetranucleotides are arranged in the table according to the 10 unique dinucleotide steps positioned in the center of the tetranucleotides. Reverse complements are also paired. For all dinucleotides and tetranucleotides, the maximum amplitude periodicities, the FVO_MAX_ and FVO_10.2_ values, the %FTS_10.2_ values, and the phase angles are listed. Additionally, within each library, each tetranucleotide and dinucleotide FVO_10.2_ was normalized by the average FVO_10.2_ of all tetranucleotides and dinucleotides, respectively.
(0.51 MB XLS)

File S3 Analysis of Fragment 67 mutations in the *in vitro* library. For all of the sequences listed in Table 1, which are 8 bp in length, graphs of the frequency profiles from the *in vitro* library are provided. Additionally, within each of these graphs, the frequency profiles of the hexamers, tetramers, and dimers centered within each octamer sequence are given. All of the profiles were normalized by their average frequencies. The occurrences of the reverse complements of all of the sequences that are non-palindromic in Table 1 were added to the frequency profiles.
(0.25 MB DOC)
