# Supplementary figures and images for: Oligonucleotide Sequence Motifs as Nucleosome Positioning Signals
Source: PLoS One. 2010 Jun 3;5(6):e10933. doi: 10.1371/journal.pone.0010933 (PMC2880596; doi:10.1371/journal.pone.0010933)

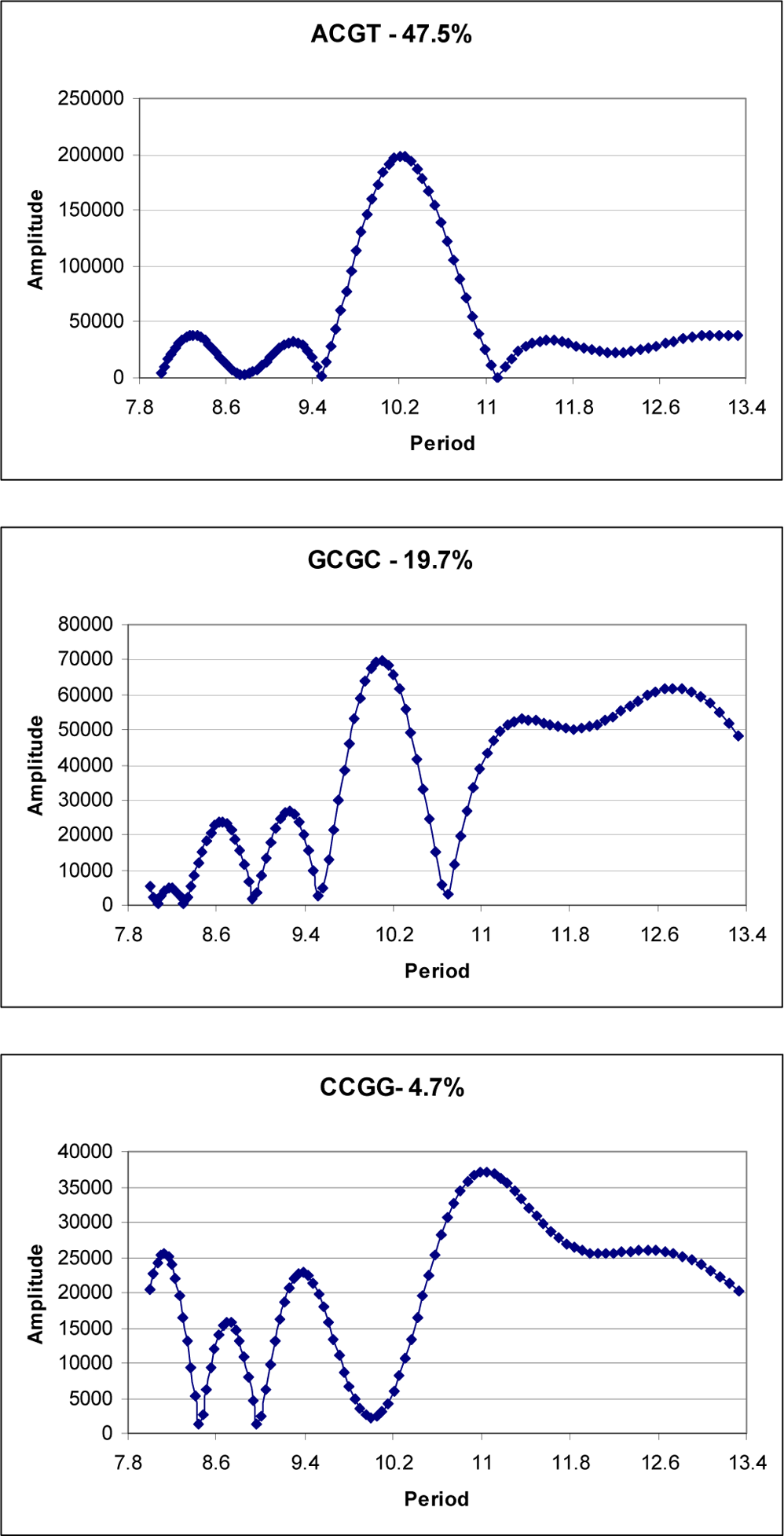

Supplement: Figure S1 — Elimination of tetranucleotides with weak periodicities. Only tetranucleotides that displayed significant ∼10 bp periodicities in the Kaplan et al. 2009 in vitro library were used for the development of the tetranucleotide consensus sequences. This was determined from the %FTS10.2 values, which represent the percent area under the Fourier-transform spectra (%FTS) from 9.8 to 10.6 bp over the area under the entire FT spectra of 8 to ∼13.5 bp. Tetranucleotides were included if they had %FTS10.2 values greater than 19% and FVO10.2 values greater than one-half standard deviation below the mean FVO10.2. Examples of tetranucleotides that display strong, borderline and weak FT spectra are shown in the figure. About 30% of the tetranucleotides were eliminated because of low %FTS10.2 scores. A high correlation was exhibited between the FVO10.2 and %FTS10.2 values (r = 0.79). Consequently, the cutoff point of a 19% FTS10.2 value eliminated nearly all of the tetranucleotides with FVO10.2 values that were less than 0.051, which was one-half standard deviation below the mean FVO10.2. Only a few tetranucleotides with %FTS10.2 values less than 19% had FVO10.2 values greater than 0.051. An exception was made for the inclusion of CTGA/TCAG into Figure 1 because its frequency profile was periodic in the central region (File S1). (0.27 MB TIF) [file pone.0010933.s001.tif]

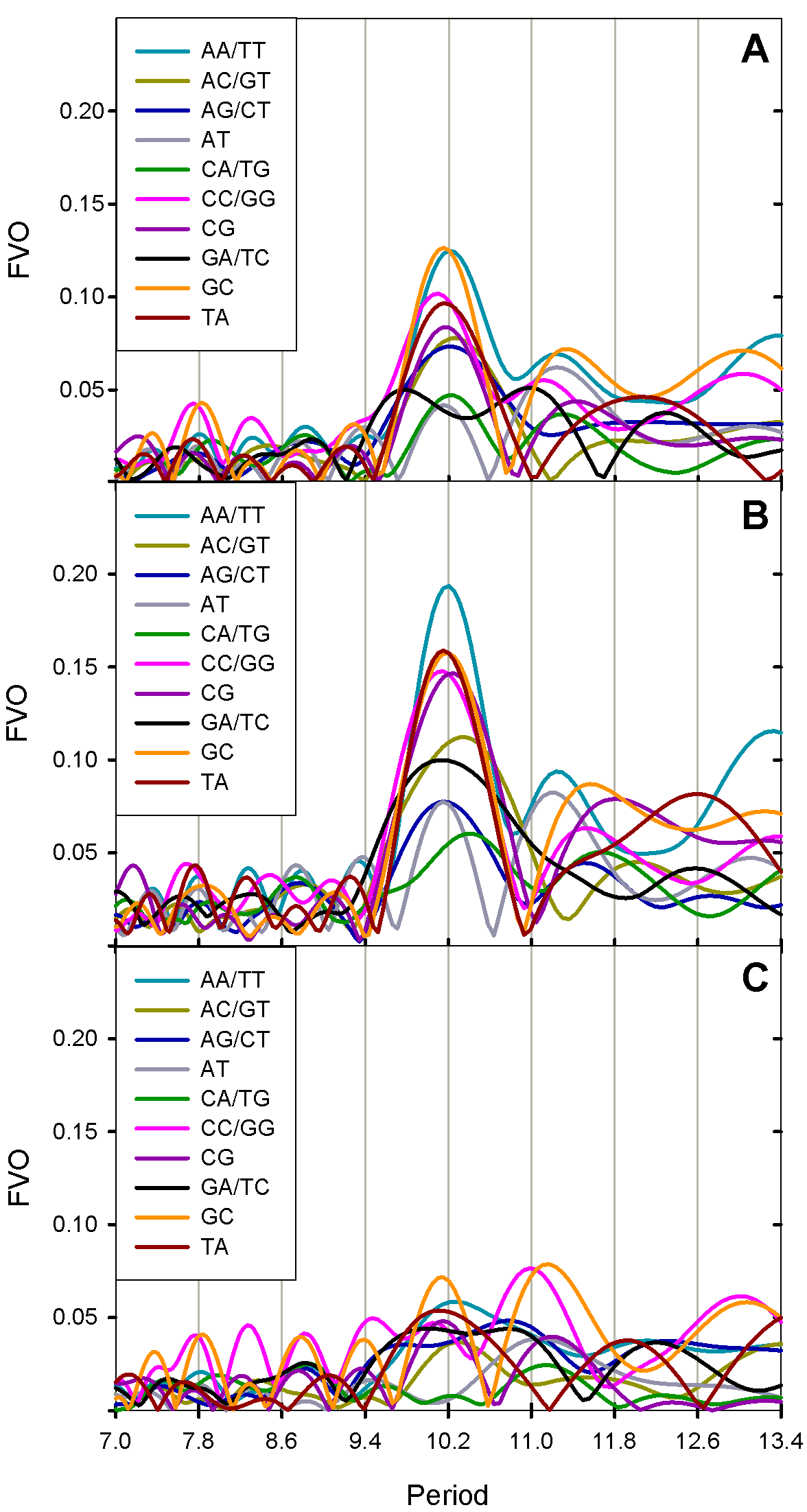

Supplement: Figure S2 — Contribution of the consensus tetranucleotides to the periodicities of the 10 unique dinucleotides. The Kaplan et al. 2009 in vitro replicate 1 library [12] was modified as described in the Methods Section in two different ways in order to evaluate the significance of the consensus tetranucleotides. Panels A, B, and C present graphs of FVO versus period for the 10 unique tetranucleotides for the Unmodified, Consensus Only, and No Consensus libraries, respectively, for sequences with greater than six reads. (0.20 MB TIF) [file pone.0010933.s002.tif]

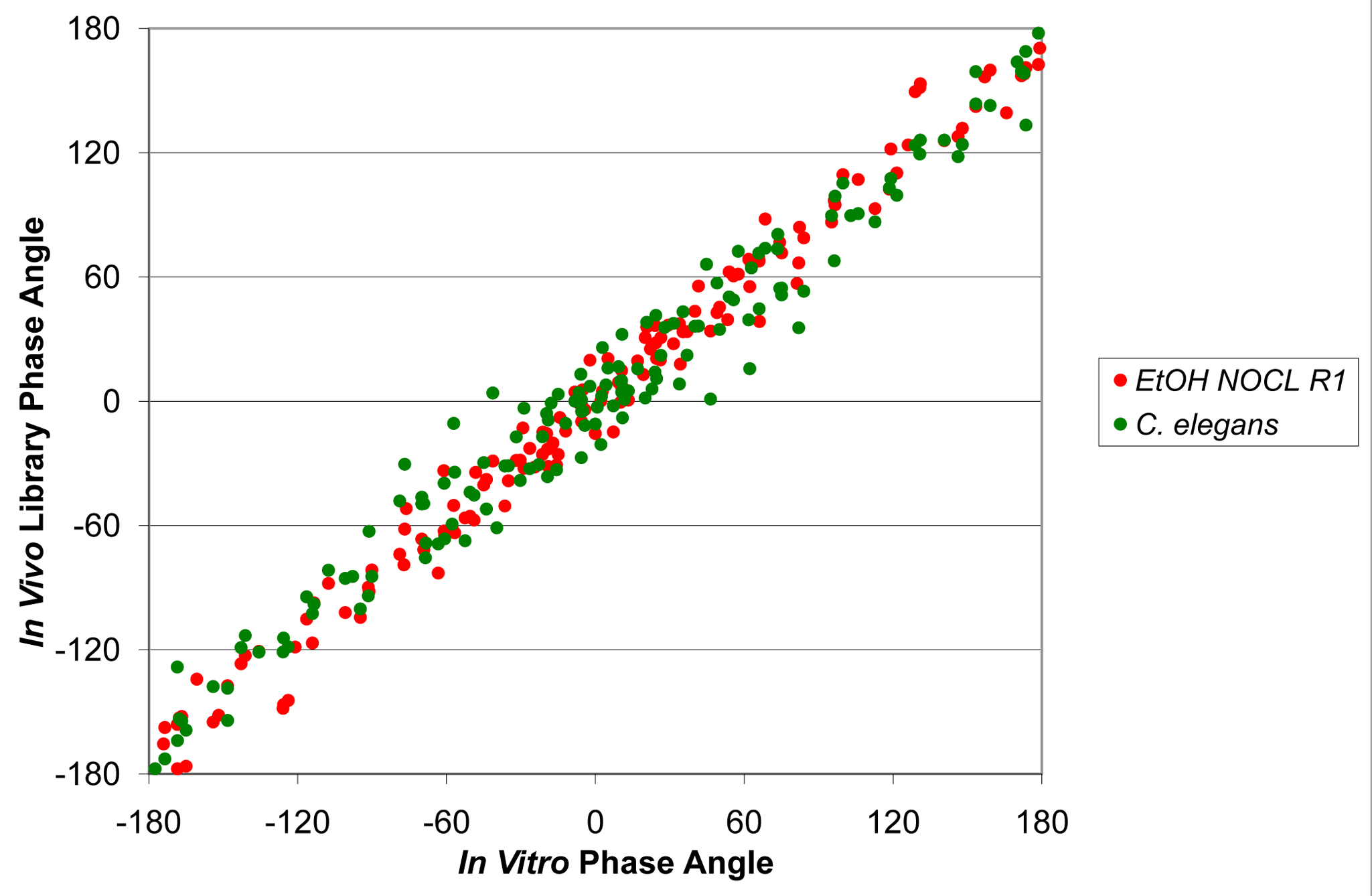

Supplement: Figure S3 — Phase angle plot (C. elegans and EtOH NOCL R1 vs. In Vitro R1). The frequency profiles of tetranucleotides from all the sequences in the in vitro replicate 1, the EtOH non-crosslinked replicate 1, and the C. elegans nucleosomal DNA sequence libraries were examined in order to calculate the phase angle for each tetranucleotide. The phase angles of the two in vivo libraries, EtOH non-crosslinked replicate 1 and C. elegans, were plotted against the in vitro replicate 1 library, yielding Pearson correlation coefficients of 0.99 and 0.98, respectively. For this phase angle correlation, approximately 30% of the phase angles from each of these three libraries were omitted because their corresponding FVO10.2 values were less than one-half standard deviation below the mean. (0.26 MB TIF) [file pone.0010933.s003.tif]

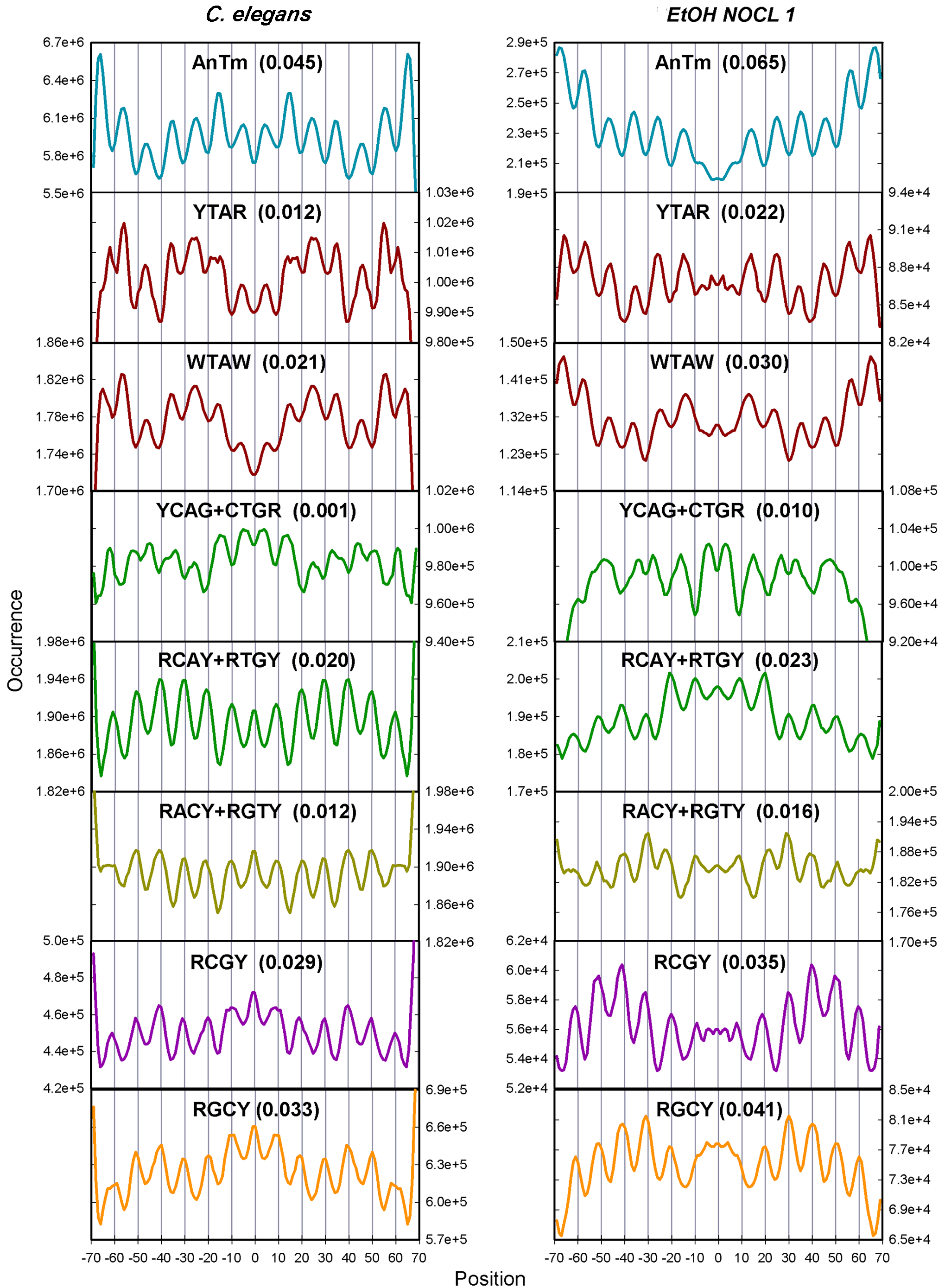

Supplement: Figure S4 — Tetranucleotide consensus sequence profiles of select in vivo libraries. The frequency profiles of the tetranucleotide consensus sequences are displayed for the Kaplan et al. 2009 ethanol non-crosslinked replicate 1 and the Valouev et al. 2008 (C. elegans) nucleosome occupancy experiments. (2.24 MB TIF) [file pone.0010933.s004.tif]

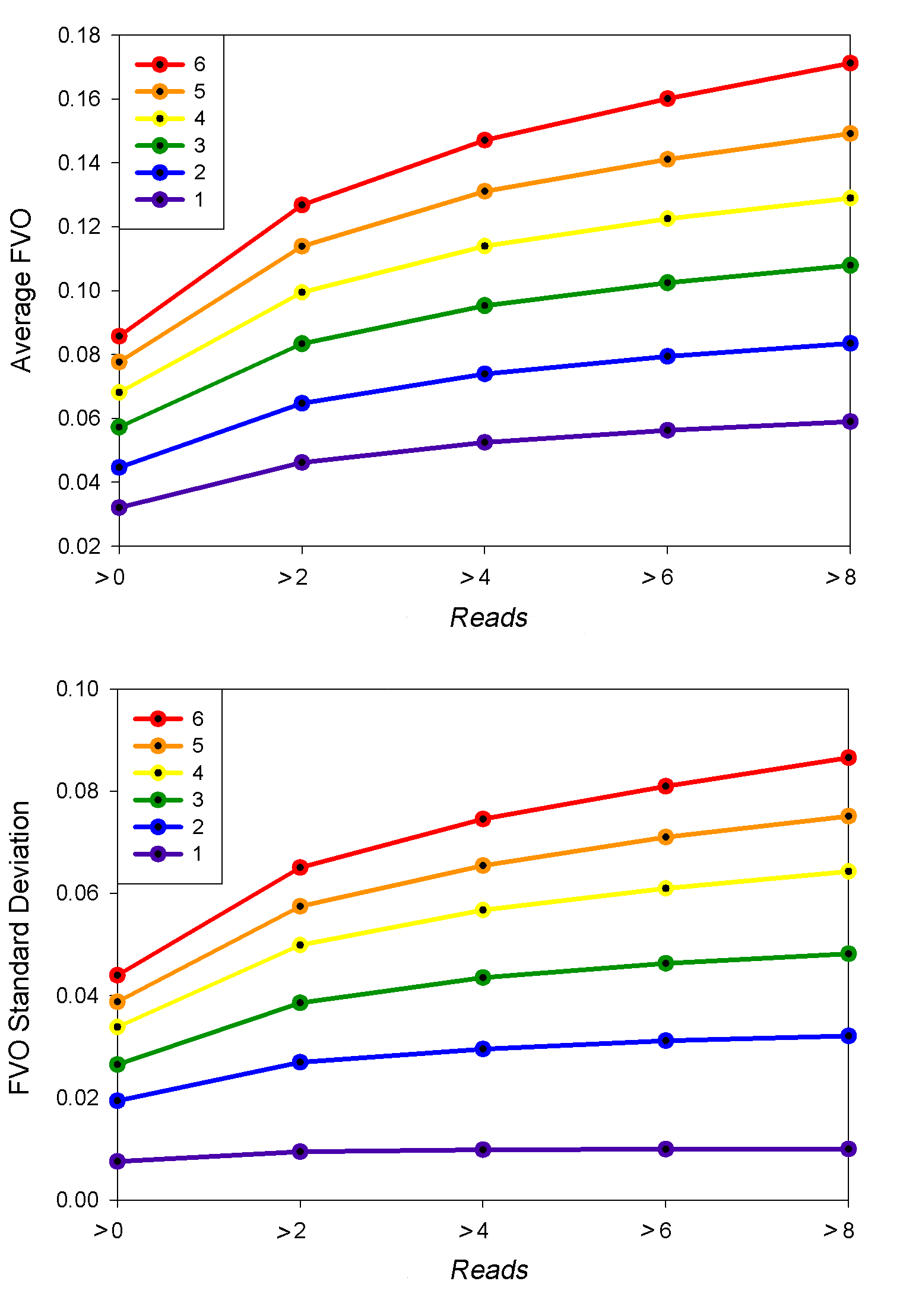

Supplement: Figure S5 — FVO analysis of motifs with different lengths. For the In Vitro Replicate 1 library, the FVO10.2 values were determined for nucleotide sequence motifs that ranged in length from 1–6 nucleotides in order to study the relationship between sequence length and enrichment of 10.2 bp periodic sequences in sub-libraries with increasing numbers of reads. The mean FVO10.2 values for each sequence length are plotted against the number of reads in the sub-libraries. The SD for each point ranged from +/−40–60% of the means. The results show that the mean FVO10.2 for each sequence length increased as function of the number of reads, and that the longer sequences increased to a greater extent than the shorter ones. Due to this observation, all nucleosomal DNA sequences were weighted by the number of reads in this study. Randomized subsets of the total library did not increase the FVO10.2 values, which indicate that the smaller number of sequences in the higher-read libraries are not causing the increases in the FVO10.2 values (data not shown). (0.11 MB TIF) [file pone.0010933.s005.tif]

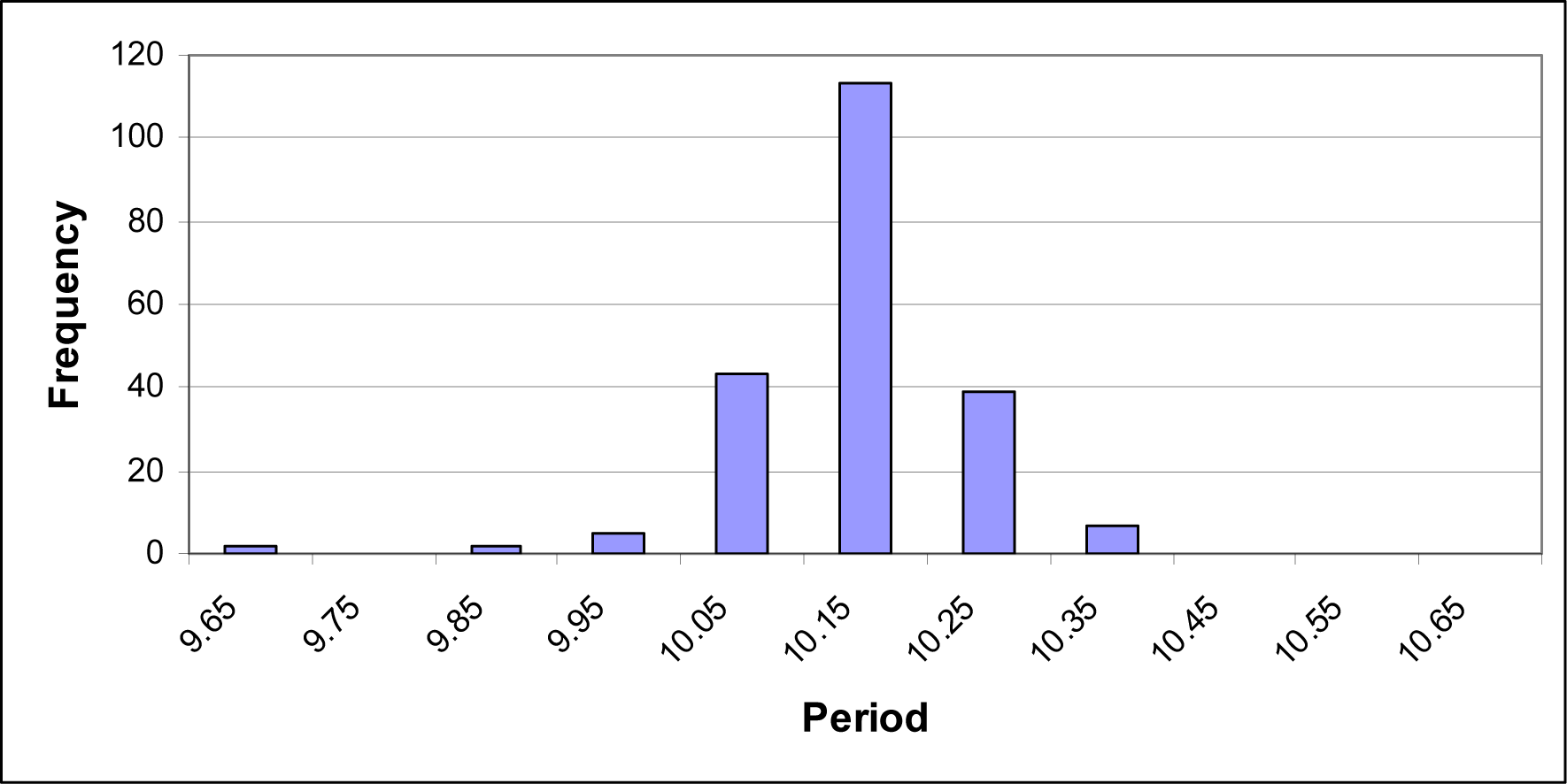

Supplement: Figure S6 — Tetranucleotide periodicities in the in vitro library. From the Fourier-transform spectra of each tetranucleotide, the maximum amplitude period over a range of 8 to ∼13.5 bp was determined for each tetranucleotide in the in vitro library. A histogram with bin widths of 0.1 bp over a range of 9.65 bp to 10.75 bp is displayed below and shows that the majority of the tetranucleotide maximum amplitude periods are near 10.2 bp. (0.08 MB TIF) [file pone.0010933.s006.tif]
