# Supplementary material for: Oligonucleotide Sequence Motifs as Nucleosome Positioning Signals
Source: PLoS One. 2010 Jun 3;5(6):e10933. doi: 10.1371/journal.pone.0010933 (PMC2880596; doi:10.1371/journal.pone.0010933)
Supplement: Table S3 — Analysis of the tetranucleotide consensus sequences for the nucleosome libraries. The maximum amplitude periodicities, the FVOMAX and FVO10.2 values, and the phase angles are displayed below for the tetranucleotide consensus sequences in the four in vivo libraries as well as the in vitro library listed in Table 2. It is important to note that due to the fact that the reverse complement pairs of dinucleotides and tetranucleotides possess opposite phase angles, the sum of the frequency profiles of reverse complement pairs will always possess phase angles of 0 or +/−180 degrees. If dinucleotides or tetranucleotides within a reverse complement pair are far from 0 or +/−180 degrees, the corresponding FVO of the reverse complement pair will decrease relative to the FVOs of the single components. On the other hand, if dinucleotides or tetranucleotides within a reverse complement pair are close to 0 or +/−180 degrees, the corresponding FVO of the reverse complement pair will reflect the FVOs of the single components. If a perfect reference point had been utilized in calculating the phase angles, the table would display 0's and +/−180's instead of −177.5's and 2.5's. (0.06 MB DOC) [file pone.0010933.s009.doc]

**Table S3. Analysis of the Tetranucleotide Consensus**

**Sequences for the Nucleosome Libraries**

| ***Cons.*** | ***Period*** | ***FVO Max*** | ***FVO 10.2*** | ***Phase ϕ*** |
| --- | --- | --- | --- | --- |
|  |  |  |  |  |
| ***In Vitro* R1** |  |  |  |  |
| antm | 10.20 | 0.1618 | 0.1618 | -177.5 |
| racyrgty | 10.05 | 0.0545 | 0.0519 | 2.5 |
| rcayrtgy | 10.20 | 0.0775 | 0.0775 | 2.5 |
| rcgy | 10.15 | 0.0970 | 0.0965 | 2.5 |
| rgcy | 10.15 | 0.1287 | 0.1268 | 2.5 |
| wtaw | 10.15 | 0.0753 | 0.0750 | -177.5 |
| ycagctgr | 10.05 | 0.0424 | 0.0409 | -177.5 |
| ytar | 10.10 | 0.0797 | 0.0783 | -177.5 |
| **EtOH NOCL R1** |  |  |  |  |
| antm | 10.31 | 0.0665 | 0.0647 | -177.5 |
| racyrgty | 10.00 | 0.0156 | 0.0139 | 2.5 |
| rcayrtgy | 10.20 | 0.0231 | 0.0231 | 2.5 |
| rcgy | 10.15 | 0.0347 | 0.0346 | 2.5 |
| rgcy | 10.20 | 0.0407 | 0.0407 | 2.5 |
| wtaw | 10.26 | 0.0302 | 0.0302 | -177.5 |
| ycagctgr | 10.99 | 0.0104 | 0.0085 | -177.5 |
| ytar | 10.10 | 0.0218 | 0.0209 | -177.5 |
| **H3H4** |  |  |  |  |
| antm | 10.10 | 0.0516 | 0.0499 | -177.5 |
| racyrgty | 9.95 | 0.0139 | 0.0094 | 2.5 |
| rcayrtgy | 10.10 | 0.0164 | 0.0155 | 2.5 |
| rcgy | 10.10 | 0.0423 | 0.0397 | 2.5 |
| rgcy | 10.00 | 0.0490 | 0.0409 | 2.5 |
| wtaw | 10.05 | 0.0396 | 0.0353 | -177.5 |
| ycagctgr | 12.12 | 0.0098 | 0.0018 | -177.5 |
| ytar | 9.22 | 0.0208 | 0.0175 | -177.5 |
| **RPO21_0** |  |  |  |  |
| antm | 10.15 | 0.0329 | 0.0322 | -177.5 |
| racyrgty | 9.95 | 0.0136 | 0.0090 | 2.5 |
| rcayrtgy | 10.05 | 0.0135 | 0.0118 | 2.5 |
| rcgy | 10.05 | 0.0279 | 0.0233 | 2.5 |
| rgcy | 10.00 | 0.0287 | 0.0225 | 2.5 |
| wtaw | 10.00 | 0.0196 | 0.0138 | -177.5 |
| ycagctgr | 10.70 | 0.0089 | 0.0024 | -177.5 |
| ytar | 10.00 | 0.0156 | 0.0124 | -177.5 |
| ***C. elegans*** | |  |  |  |
| antm | 10.05 | 0.0482 | 0.0445 | -177.5 |
| racyrgty | 9.95 | 0.0166 | 0.0123 | 2.5 |
| rcayrtgy | 10.00 | 0.0245 | 0.0199 | 2.5 |
| rcgy | 10.00 | 0.0373 | 0.0290 | 2.5 |
| rgcy | 10.00 | 0.0407 | 0.0327 | 2.5 |
| wtaw | 10.00 | 0.0206 | 0.0160 | -177.5 |
| ycagctgr | 9.48 | 0.0082 | 0.0007 | -177.5 |
| ytar | 10.00 | 0.0153 | 0.0121 | -177.5 |
