# Supplementary material for: Oligonucleotide Sequence Motifs as Nucleosome Positioning Signals
Source: PLoS One. 2010 Jun 3;5(6):e10933. doi: 10.1371/journal.pone.0010933 (PMC2880596; doi:10.1371/journal.pone.0010933)
Supplement: Table S4 — Analysis of the periodicities for profiles in figure 8. The average frequencies, maximum amplitude periodicities, and FVO10.2 values are displayed for select motifs derived from the A) Kaplan et al. 2009 in vitro library, B) the Kaplan et al. 2009 EtOH non-crosslinked library, C) the Mavrich et al. 2008 library, D) the Weiner et al. 2009 library, and E) the Valouev et al. 2008 C. elegans library. NS = Not Significant. (0.04 MB DOC) [file pone.0010933.s010.doc]

**Table S4. Analysis of the Periodicities**

**for Profiles in Figure 8.**

|  | **Avg Freq** | **Period** | **FVO 10.2** |
| --- | --- | --- | --- |
| **A** |  |  |  |
| A_3_-A_5_+T_3_-T_5_ | 797520 | 10.26 | 0.144 |
| AA+TT | 1776272 | 10.20 | 0.070 |
| sAAs+sTTs | 359357 |  | 0.012 - NS |
| **B** |  |  |  |
| A_3_-A_5_+T_3_-T_5_ | 505705 | 10.31 | 0.056 |
| AA+TT | 1172262 | 10.26 | 0.028 |
| sAAs+sTTs | 264005 |  | 0.001 - NS |
| **C** |  |  |  |
| A_3_-A_5_+T_3_-T_5_ | 331978 | 10.15 | 0.039 |
| AA+TT | 753984 | 10.10 | 0.021 |
| sAAs+sTTs | 152517 |  | 0.000 - NS |
| **D** |  |  |  |
| A_3_-A_5_+T_3_-T_5_ | 230395 | 10.15 | 0.027 |
| AA+TT | 459291 | 10.15 | 0.014 |
| sAAs+sTTs | 82556 |  | 0.003 - NS |
| **E** |  |  |  |
| A_3_-A_5_+T_3_-T_5_ | 9817156 | 10.10 | 0.042 |
| AA+TT | 16621207 | 10.05 | 0.024 |
| sAAs+sTTs | 2369139 |  | 0.000 - NS |
