# Supplementary material for: Oligonucleotide Sequence Motifs as Nucleosome Positioning Signals
Source: PLoS One. 2010 Jun 3;5(6):e10933. doi: 10.1371/journal.pone.0010933 (PMC2880596; doi:10.1371/journal.pone.0010933)
Supplement: File S3 — Analysis of Fragment 67 mutations in the in vitro library. For all of the sequences listed in Table 1, which are 8 bp in length, graphs of the frequency profiles from the in vitro library are provided. Additionally, within each of these graphs, the frequency profiles of the hexamers, tetramers, and dimers centered within each octamer sequence are given. All of the profiles were normalized by their average frequencies. The occurrences of the reverse complements of all of the sequences that are non-palindromic in Table 1 were added to the frequency profiles. (0.25 MB DOC) [file pone.0010933.s013.doc]

**Analysis of Fragment 67 Mutations in the *In Vitro* Library (Supplemental File 4)**

For all of the sequences listed in Table 1, which are 8 bp in length, graphs of the frequency profiles from the *in vitro* library are provided. Additionally, within each of these graphs, the frequency profiles of the core hexamers, tetramers, and dimers centered within each octamer sequence are given. All of the profiles were normalized by their average frequencies. The occurrences of the reverse complement pairs of all of the sequences were added to the frequency profiles.

| **Construct** | **Sequence** | **∆G** | **% of 67** | **FVO (8)** | **FVO (6)** | **FVO (4)** | **FVO (2)** |
| --- | --- | --- | --- | --- | --- | --- | --- |
| 67 | CTCTAGAG | 0 | 100 | 0.256 | 0.149 | 0.074 | 0.047 |
| 67-m1 | CTCCAGAG | 586 | 85 | 0.233 | 0.111 | 0.053 | 0.021 |
| 67-m2 | CTCAAGAG | 678 | 77 | 0.143 | 0.027 | 0.005 | 0.070 |
| 67-m3 | CTCCCGAG | 956 | 69 | 0.083 | 0.046 | 0.007 | 0.042 |
| 67-m4 | CTCATGAG | 1195 | 61 | 0.079 | 0.038 | 0.055 | 0.027 |
| 67-m5 | CTCGAGAG | 974 | 69 | 0.018 | 0.010 | 0.034 | 0.010 |
| 67-m6 | CTCTCGAG | 1129 | 61 | 0.018 | 0.034 | 0.034 | 0.010 |
|  |  |  |  |  |  |  |  |
| 67-m7 | CTGTAGAG | 556 | 84 | 0.139 | 0.080 | 0.026 | 0.047 |
| 67-m8 | CTCTACAG | 556 | 82 | 0.139 | 0.080 | 0.026 | 0.047 |
| 67-m9 | CTGTACAG | 761 | 68 | 0.031 | 0.009 | 0.018 | 0.047 |
| 67-m10 | CAGTACTG | 1130 | 58 | 0.100 | 0.059 | 0.018 | 0.047 |
|  |  |  |  |  |  |  |  |
| 601+25 | TGCTAGAG | 31 | 96 | 0.143 | 0.105 | 0.074 | 0.047 |
| 601-39 | GACTAGGG | 92 | 83 | 0.130 | 0.084 | 0.074 | 0.047 |
| 5S-16 | CTTTAAAT | -140 | 93 | 0.156 | 0.144 | 0.086 | 0.047 |
| 601-16 | GGTTAAAA | 107 | 80 | 0.146 | 0.107 | 0.086 | 0.047 |
| 5S-7 | GCTTAACT | 171 | 83 | 0.066 | 0.077 | 0.086 | 0.047 |
| 601+16 | GTTTAAGC | -247 | 88 | 0.183 | 0.124 | 0.086 | 0.047 |
